# Enrofloxacin treatment alters cecal microbiota and metabolites and selects for fluoroquinolone-resistant *Campylobacter jejuni* in chickens

**DOI:** 10.1128/spectrum.01587-26

**Published:** 2026-09-17

**Authors:** Jinji Pang, Qijing Zhang, Orhan Sahin

**Affiliations:** 1 Department of Veterinary Microbiology and Preventive Medicine, College of Veterinary Medicine, Iowa State University 70724 , Ames, Iowa, USA; 2 Department of Statistics, Iowa State University 231411 , Ames, Iowa, USA; 3 Department of Veterinary Diagnostic and Production Animal Medicine, College of Veterinary Medicine, Iowa State University 70724 , Ames, Iowa, USA; University of Georgia College of Veterinary Medicine, Athens, Georgia, USA

**Keywords:** *Campylobacter*, fluoroquinolone, antibiotic resistance, broilers, gut microbiota, metabolome

## Abstract

*Campylobacter* is a leading cause of foodborne illness and is commonly present in the poultry gut. Fluoroquinolone (FQ)-resistant *Campylobacter* quickly emerges in chickens infected with FQ-susceptible *Campylobacter* and treated with an FQ antibiotic via drinking water due to selection and expansion of FQ-resistant mutants. However, it is unclear whether FQ treatment alters the gut microbiota and metabolome, and consequently provides a favorable environment for FQ-resistant *Campylobacter* to rapidly expand in the intestine. Here, we investigated cecal microbiota and metabolome profiles of broiler chickens infected with *Campylobacter jejuni* and treated with enrofloxacin (ENRO). Although ENRO treatment initially reduced *Campylobacter* colonization, rapid selection and expansion of FQ-resistant *C. jejuni* mutants followed in treated birds. 16S rRNA gene sequencing revealed significant decreases in the relative abundance of phylum *Actinobacteriota* and several genera, including *Proteus*, *Blautia*, *Romboutsia*, *Erysipelotrichaceae_uncultured*, *Candidatus_Arthromitus*, *Weissella*, *Butyricicoccus*, and *Clostridia*_*vadinBB60_group*, in ENRO-treated birds. Untargeted metabolomics analyses indicated notable differences in amino acid, lipid, carbohydrate, cofactor, vitamin, and xenobiotic metabolites between ENRO-treated and control groups. Interestingly, the ENRO-treated group had a significantly higher concentration of phenethylamine. Correlation analysis found several significantly correlated pairs of bacterial taxa and metabolomic features. These results indicate that gut microbiota diversity, composition, and metabolites were significantly altered by ENRO treatment, which may facilitate the rapid propagation of FQ-resistant *Campylobacter* in the chicken gut. Future studies are needed to identify specific microbiota members and metabolites linked to the emergence and proliferation of FQ resistance, ultimately aiding the development of antibiotic resistance mitigation strategies.

**IMPORTANCE:**

*Campylobacter* is a major foodborne bacterium often linked to poultry, and antibiotic-resistant strains can make infections harder to treat. This study shows that treating chickens with enrofloxacin, an antibiotic in the fluoroquinolone family, quickly selected for resistant *Campylobacter* and also changed the gut bacterial community and chemical makeup of the chicken intestine. These changes may help resistant *Campylobacter* grow and persist after treatment. By linking antibiotic treatment with changes in gut bacteria and metabolites, this work provides a broader view of how antimicrobial use can unintentionally promote resistance in food animals. The findings highlight the need to consider the whole gut environment when designing strategies to limit antibiotic resistance and improve poultry-related food safety.

## INTRODUCTION

*Campylobacter* is the leading bacterial cause of foodborne gastroenteritis worldwide and a major public health concern (1–3). The organism has a broad animal reservoir and colonizes the gastrointestinal (GI) tracts of a wide range of species, including poultry, cattle, pigs, dogs, cats, and wild animals (4). Transmission of *Campylobacter* to humans primarily occurs via the foodborne route, by intake of contaminated food and water (4, 5). Among all the animal species, poultry is recognized as the major reservoir of *Campylobacter*, and consumption of raw or undercooked poultry meat is a main risk factor for human campylobacteriosis (6–8). Common campylobacteriosis symptoms include fever, stomach discomfort, and watery or bloody stools (9). Severe complications, including Guillain-Barré syndrome, reactive arthritis, and irritable bowel syndrome, can also occur following initial *Campylobacter* infection (10, 11). Among the species implicated in campylobacteriosis, *Campylobacter jejuni* is the primary species responsible for foodborne diarrhea in humans (10, 12).

Although the majority of *Campylobacter* infections are self-limiting, antibiotic treatment is often employed to treat severe cases and infections in immunocompromised patients (10, 11, 13). Fluoroquinolones (FQs), such as ciprofloxacin, and macrolides (e.g., erythromycin) are among the most often recommended antibiotics for clinical treatment of human campylobacteriosis (10, 11, 13). However, many *Campylobacter* strains have become resistant to both classes of antimicrobials, especially FQs, reducing the effectiveness of clinical therapy (14). The emergence of FQ-resistant *Campylobacter* is likely due to use of FQ antibiotics in both animal agriculture and human clinical settings, and its rise in prevalence in the United States is correlated with the introduction of FQs for use in poultry in the mid-1990s (15–17). For example, enrofloxacin (ENRO), a veterinary antibiotic derived from ciprofloxacin, shares analogous mechanisms of action with ciprofloxacin yet possesses separate approved applications and formulations in animals (16). The use of ENRO to treat respiratory disease in poultry was clearly shown to cause the development and spread of FQ-resistant *Campylobacter* (18). Additionally, multiple studies demonstrated the rapid selection of FQ-resistant *Campylobacter* in chickens treated with ENRO (18–23). Given the importance of poultry in transmitting foodborne campylobacteriosis, the Food and Drug Administration withdrew approval for FQ antibiotics from poultry production in the United States in 2005 (24). Besides poultry, FQ-resistant *Campylobacter* is also detected in other animal species, including ruminants and wildlife (17).

FQs inhibit bacterial growth by specifically targeting two essential enzymes in bacteria, namely DNA gyrase and topoisomerase IV, which play a vital role in the replication of bacterial DNA (25). In FQ-resistant *Campylobacter*, the resistance is mainly mediated by point mutations in the quinolone resistance-determining region of the A subunit of DNA Gyrase (GyrA), one of the two subunits in bacterial DNA gyrase (26). Mutations at positions Thr-86, Asp-90, and Ala-70 in GyrA have been associated with FQ resistance in *Campylobacter*, and the Thr-86-Ile substitution has been identified as the primary cause of high-level resistance to FQs (27–29). In addition to point mutations in GyrA, the CmeABC multidrug efflux pump has also been identified as a key mechanism for FQ resistance in *Campylobacter* by reducing antibiotic accumulation in bacterial cells (27–29). According to the most recent antibiotic resistance threats report from the Centers for Disease Control and Prevention, drug-resistant *Campylobacter* is one of the serious threats in the United States, and 29% of *Campylobacter* from clinical infections had decreased susceptibility to FQs or macrolides (30).

The microbial community residing in the GI tract of chickens is important for the overall gut health and the host’s ability to resist pathogens (31–34). The microbiota aids in breaking down complex dietary components that enhance nutrient absorption for the host and produces essential metabolites, such as short-chain fatty acids (SCFAs) (31–33). Moreover, gut microbiota contributes to the development and maturation of the immune system, influencing the chicken’s ability to recognize and combat various pathogens by synthesizing antimicrobial compounds, such as bacteriocins, SCFAs, and hydrogen peroxide (31). By occupying ecological niches and competing with pathogenic bacteria, members of beneficial gut microbiota in chickens also reduce the opportunity for the colonization of pathogenic foodborne bacteria, thereby reducing the risk of carcass contamination and subsequent transmission to humans (31, 35).

Antibiotic treatment is known to disrupt the gut microbiota in animals. We recently examined the effects of FQ treatments on gut microbiota in calves orally inoculated with *C. jejuni* and found that subcutaneous FQ treatment not only altered the gut microbiota diversity in calves but also changed the correlation pattern between *Campylobacter* and other bacterial taxa present in the gut (36, 37). Plata et al. also investigated the effect of four (bacitracin methylene disalicylate, avilamycin, virginiamycin, and narasin) antimicrobial growth promoters (AGPs) on broiler gut microbiome changes and found that different AGPs led to distinct cecal microbial community structures (38). In addition, a number of studies assessed changes in the gut metabolome during antibiotic treatment. For example, virginiamycin treatment altered 217 metabolites and 10 KEGG pathways in broiler ceca (39). In a laboratory setting, giving enrofloxacin through oral gavage for 5 consecutive days also affected broilers’ cecal microbiota, metabolome, and antibiotic-resistance gene expressions (40). These examples indicate the importance of characterizing changes in the gut microbiota and metabolome as a result of antibiotic treatment to better understand the relationship between microbiota alteration and the development of FQ-resistant *Campylobacter*, as well as the mechanistic aspects involved in this interaction.

It is known that FQ-resistant *Campylobacter* quickly develops in chickens treated with FQs via drinking water (20–23, 41). However, it remains unknown whether the antibiotic treatment-mediated alteration of gut microbiota and metabolome in the intestine creates a favorable environment for the rapid expansion of FQ-resistant *Campylobacter*. To address this question, broiler chickens were orally challenged with *C. jejuni* and treated with ENRO in drinking water in the current study. 16S rRNA gene sequencing, shotgun metagenomics, and untargeted metabolomics profiling of cecal contents were performed to analyze microbiota composition and metabolite profiles in response to the ENRO treatment and in relation to FQ-resistance development dynamics in *Campylobacter* in the chicken gut.

## MATERIALS AND METHODS

### Bacterial strain and culture conditions

*Campylobacter jejuni* W7, which is a highly motile derivative of strain NCTC 11168, was used in this study (42). The strain was routinely grown on Mueller-Hinton (MH) agar (Becton, Dickinson and Company, MD, USA) at 42°C under microaerobic conditions (85% N_2_, 10% CO_2_, 5% O_2_). For preparation of inoculum used in the chicken study (see below), the bacterial growth from the frozen stock on the primary MH agar was passaged to new MH agar plates and incubated to the late logarithmic phase (approximately 16–18 h). The bacterial growth on the agar plate was then harvested in sterile MH broth (Becton, Dickinson and Company), and the OD_600_ was adjusted to 0.01, which equaled a final concentration of approximately 1 × 10^7^ CFU/mL, as determined by viable bacterial count. The motility of the isolate was confirmed using MH medium containing 0.4% Bacto Agar (Becton, Dickinson and Company) as described in a previous publication (43).

### Animal study design and sample collection

The Iowa State University Institutional Animal Care and Use Committee (IACUC) approved all the procedures and protocols for animal experiments prior to the start of the study. All methods and procedures for this animal study fully complied with the Committee’s guidelines and regulations as appropriate.

Ninety 1-day-old broiler chicks (mixed sex) were obtained from a commercial hatchery and housed at the laboratory animal facilities of Iowa State University. The chicks were placed on new wood shavings in floor pens. Water and commercial non-medicated broiler feed were given *ad libitum* throughout the trial. At 5 days of age, all birds were confirmed to be *Campylobacter*-free by culturing the cloacal swabs on MH agar supplemented (MH+SS) with *Campylobacter* growth supplement (SR084E; Oxoid, Basingstoke, England) and modified Preston *Campylobacter* selective supplement (SR0204E; Oxoid). At 7 days of age, 10 birds were randomly selected and euthanized, and their cecal contents were collected to serve as the microbiota and metabolome baseline group. The remaining chickens were orally inoculated with a freshly prepared culture of *C. jejuni* W7 (200 μL of 10^7^ CFU/mL per bird) before being randomly divided into an ENRO treatment group and a control group (40 birds/group). At 12 days of age, cloacal swabs were collected from all the birds and cultured on the selective agar plates (MH+SS) to confirm their *Campylobacter* colonization status. At 14 days of age, chickens in the ENRO treatment groups were administered ENRO (Sigma-Aldrich Co. LLC., USA) in the drinking water for 5 consecutive days. The ENRO was first dissolved in dimethyl sulfoxide (DMSO) and then diluted with tap water to achieve a final concentration of 50 ppm (50 mg ENRO in 1 L water). Conversely, the control group received non-antibiotic-supplemented drinking water containing an equivalent amount of DMSO (5 mL DMSO in 1 L water). Water treatments were given each day with daily fresh preparations. Ten birds from each group were euthanized and necropsied on days 2, 7, 12, and 17 post-initiation of treatment (DPT-2, -7, -12, and -17); the cecal contents were collected and processed for multiple purposes as described in the following sections. Major activities performed during the entire study are summarized in Table 1.

**TABLE 1:** Summary of activities performed over the 31-day duration of the animal study starting with day-old commercial broilers

Activities	Pre-treatment				Treatment			Post-treatment			Groups^*a*^
	Day 1	5	7	12	14–15	16	17–18	21	26	31	
Birds arrived											Baseline ENRO control
Pre-inoculation cloacal swab											Baseline ENRO Control
Necropsy and cecal content collection											Baseline
*C. jejuni* challenge (oral)											ENRO Control
Post-inoculation cloacal swab											ENRO Control
ENRO treatment (days 14–18)											ENRO
Necropsy and cecal content collection											ENRO Control

^*a*^ Groups: baseline group (*n* = 10 birds), ENRO treatment group (*n* = 40 birds), control group (*n* = 40 birds). Each shaded box indicates the day when the respective activity was conducted.

### Enumeration of *Campylobacter*

Cecal contents were collected at necropsy from each bird and kept on ice until processed further. Soon after the necropsy, cecal contents were serially diluted with sterile MH broth. An inoculum of 100 μL from appropriate dilutions was plated onto MH+SS agar plates and MH+SS agar plates plus ciprofloxacin (4 μg/mL) for enumerating (CFU/g) total *Campylobacter* and FQ-resistant *Campylobacter*, respectively. Plates were incubated in a microaerobic environment at 42°C for up to 3 days. A single *Campylobacter*-like colony from each presumptive positive sample was collected and later confirmed to be *C. jejuni* by MALDI-TOF mass spectrometry, following the manufacturer’s (Bruker Daltonik, Billerica, MA, USA) operating procedures at ISU Veterinary Diagnostic Laboratory. The final CFU/g of cecal content was calculated based on the colony counts and the dilution factors used.

### DNA extraction, 16S rRNA gene sequencing, and shotgun metagenomics

Microbial DNA was extracted from the 90 cecal samples using the ZymoBIOMICS kit (Zymo Research, Irvine, CA, USA) following the manufacturer’s instructions. Briefly, approximately 200 mg of cecal content from each sample was transferred to a 2 mL ZR BashingBead lysis tube immediately following collection at necropsy and without freezing. Each lysis tube was then filled with 250 µL deionized sterile water, 750 µL lysis solution, and 50 µL (from 20 mg/mL stock) proteinase K (Qiagen, Germantown, MD, USA). After being processed by a bead beater for 10 min, the lysis tubes were incubated for at least 30 min at 55°C, centrifuged at 10,000 × *g* for 3 min, and the resultant supernatant was collected and transferred to a collection tube. Washing was done with DNA Wash Buffers 1 and 2, and the end product was eluted using DNase/RNase-free water. The concentration of the eluted DNA was assessed using a NanoDrop 3300 Fluorospectrophotometer (Nanodrop Technologies, Wilmington, DE, USA) initially and later confirmed using a Qubit fluorometer (Turner BioSystems, Sunnyvale, CA, USA). After normalization, all the DNA extracts were transferred to 96-well microtiter plates and submitted for both 16S rRNA gene sequencing and shotgun metagenomics sequencing at the DNA Facility of Iowa State University using the Illumina MiSeq platform (with 2 × 250 paired-end technology) in a single flow cell lane or the Illumina NovaSeq 6000 platform (with 2 × 250 paired-end technology) in four flow cell lanes, respectively. The sequencing followed the Earth Microbiome Project protocol (44).

### Sample preparation and metabolomics analysis

Approximately 100 mg of cecal content was collected from each bird and placed into a pre-chilled polypropylene tube (provided by Metabolon) during the necropsy. The samples were then flash-frozen using dry ice and stored at −80°C. The samples were shipped on dry ice to Metabolon, Inc. (Morrisville, NC, USA). Untargeted metabolomics profiling of the cecal samples through an ultra-high-performance liquid chromatography-tandem mass spectrometry (UPLC-MS/MS) was performed by Metabolon, Inc., as previously described (45).

### Statistical analysis

After the log10 transformation, the *Campylobacter* colony counts in cecal contents were analyzed using non-parametric tests. The Wilcoxon and Kruskal-Wallis tests were used to compare the difference in *Campylobacter* colonization levels among different groups. The 16S rRNA gene sequence data were analyzed through the QIIME 2 pipeline using bioinformatic methods (46). First, the raw sequencing data from the DNA facility were demultiplexed, then denoised by the DADA2 method to remove noise and chimeric sequences, and similar sequences were clustered into amplicon sequence variants (ASVs) (47). Taxonomy classification of ASVs was performed by comparing them to the SILVA 16S rRNA gene database (48, 49). To assess the effect of ENRO treatment on the diversity and composition of cecal microbiota, alpha and beta diversity metrics were calculated on the QIIME 2 platform (46). For alpha diversity, the richness was estimated using observed features, and the evenness was determined using the Shannon index and Pielou’s evenness index. The phylogenetic diversity was evaluated by Faith’s phylogenetic diversity (Faith’s pd) index. Beta diversity analysis was conducted using unweighted UniFrac distance, which quantitatively measures dissimilarity between microbial communities considering their phylogenetic relationships. The resulting distance matrix was visualized using a two-dimensional principal coordinate analysis (PCoA) plot. Analysis of similarity (ANOSIM) was conducted in R using the vegan package to further confirm the beta diversity analysis results (50). Analysis of the compositions of microbiomes with bias correction (ANCOM-BC) was used to identify differentially abundant phyla and genera between the ENRO treatment group and the control group (51). For shotgun metagenomics, MEGAHIT (v1.2.9) from KBase was used to assemble the short reads into metagenomes with default parameters (52). RASTtk (v1.073) was used to further annotate genome assemblies and assign functions for genomes (52). Taxonomy classification of metagenomic reads was assigned using the Kraken 2 (v2.1.2) database (53). In relation to metabolomics findings, putative phenethylamine-associated microbial species were identified by measuring the correlation between the relative abundance of each species and phenethylamine concentration across treatments and sampling time points. Pearson’s correlation coefficient and linear mixed effects model were used to measure the correlation between candidate species and phenethylamine.

The metabolomics data were subjected to statistical analysis using scaled and imputed data provided by Metabolon, Inc. Briefly, the raw data obtained from the UPLC-MS/MS run were initially represented as peak intensities for each metabolite. Subsequently, the median of each metabolite was standardized to 1, and any missing values were imputed with the lowest observed value of that metabolite across all samples, resulting in the production of scaled imputed data. The scaled imputed data were subjected to natural logarithmic transformation for each metabolite before employing Welch’s two-sample *t*-test to detect significant differences in biochemicals between the experimental groups. Differentially abundant metabolites were defined using both an effect-size threshold and a multiple-testing-adjusted significance threshold: fold-change >2 or <0.5, equivalent to |log2 fold-change| > 1, together with *q* value < 0.05. This combined criterion was selected as a conservative screening approach to prioritize metabolites showing both substantial abundance changes and controlled false-discovery risk in untargeted metabolomics analyses. Principal components analysis (PCA) was conducted to gain a thorough and comparative view of the metabolomics data acquired from the cecal content data set. Correlation analysis of dominant bacterial genera (derived from the 16S rRNA gene sequencing) with differentially abundant metabolites was calculated using Pearson’s correlation test, also performed by Metabolon. A *P* value of <0.05 was considered significant for all statistical analyses.

## RESULTS

### Effect of enrofloxacin treatment on *C. jejuni* colonization in chicken ceca

Culturing of cloacal swabs confirmed that while all the chickens were free of *Campylobacter* prior to the inoculation, they were all colonized with *C. jejuni* on day post-inoculation (DPI)—5 (results not shown). To evaluate the effect of ENRO treatment on *C. jejuni* counts, differential plating was used to enumerate both total and FQ-resistant *Campylobacter* in cecal contents as described in our previous study (54). ENRO treatment significantly reduced total *Campylobacter* CFU counts on day post-initiation of treatment (DPT)—2, resulting in a 2.82 log_10_ unit reduction (5.99 log vs 3.17 log, *P* value = 0.017) between the control group and the ENRO-treatment group (Fig. 1A). On DPT-7, -12, and -17, the total *Campylobacter* CFUs remained high in both groups, but the *C. jejuni* colonization level on DPT-17 was higher in the ENRO treatment group than the control group (8.00 log vs 7.05 log; *P* value = 0.001). Notably, and consistent with the previous findings (20, 23), ENRO treatment promoted the development of FQ-resistant *Campylobacter*, while no FQ-resistant *Campylobacter* was detected in the control group during the entire experiment (Fig. 1B). Of particular note, the *C. jejuni* population colonizing the ENRO treatment group was nearly 100% FQ-resistant from DPT-2 to DPT-17 (Fig. 1C).

**Fig 1:**
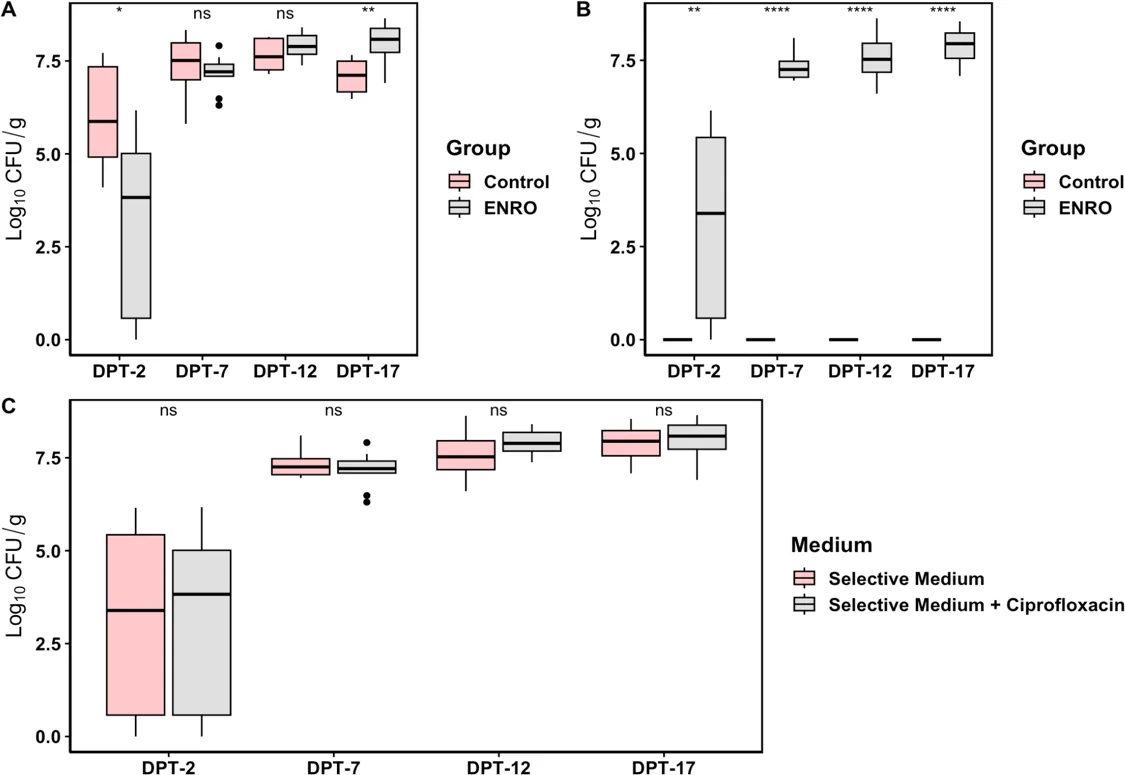
*C. jejuni* CFUs in the cecal contents of chickens following ENRO treatment. Total CFU counts obtained by *Campylobacter* selective medium (MH+SS) in the ENRO-treated group and untreated (control) group (**A**). FQ-resistant *Campylobacter* CFUs obtained by MH+SS containing ciprofloxacin (4 µg/mL) in both groups (**B**). Total *Campylobacter* CFUs (by selective medium) and FQ-resistant *Campylobacter* CFUs (by selective medium + ciprofloxacin) in the ENRO-treated group only (**C**). CFU values are log_10_ transformed, and CFU medians are shown as the middle horizontal lines, with the upper and lower bounds of the box showing the lower quartile and upper quartile of the data. DPT, day post-initiation of ENRO treatment. The *P* values were calculated using a non-parametric Wilcoxon test (****P value < 0.0001, **P value < 0.01, *P value < 0.05; ns, not significant).

### Effect of enrofloxacin treatment on cecal microbiota diversity and community structure in broilers

To determine how the ENRO treatment altered cecal microbiota in relation to the development of FQ-resistant *Campylobacter*, cecal contents from 77 birds (37 from ENRO treatment and 40 from the control) were analyzed by 16S rRNA gene profiling. Of note, the sequencing data from the baseline birds (*n* = 10) were excluded from the analyses as the differences in microbiota composition appeared to be primarily due to the age difference between the groups. In addition, the data from three birds in the ENRO group were excluded due to QC-related issues. Across all samples tested, a total of 605 ASVs were identified, with 338 ASVs having a frequency higher than 43 and thus selected for future analysis. For the diversity within samples from the same group (alpha diversity), the differences in species richness (Fig. 2A and B) and evenness (Fig. 2B and C) were minimal between the control and ENRO groups when the effect of the bird age was ignored; however, ENRO treatment caused a significant decrease in phylogenetic diversity of the cecal microbial community compared with the control group (Fig. 2D). More specifically, ENRO treatment significantly decreased the cecal microbial diversity metrics of species richness on DPT-2 and -7 (Fig. 3A and B), evenness on DPT-2 (Fig. 3B and C), and phylogenetic diversity on DPT-2 and -7 (Fig. 3D) when samples were grouped by treatment status and sampling days (Fig. 3).

**Fig 2:**
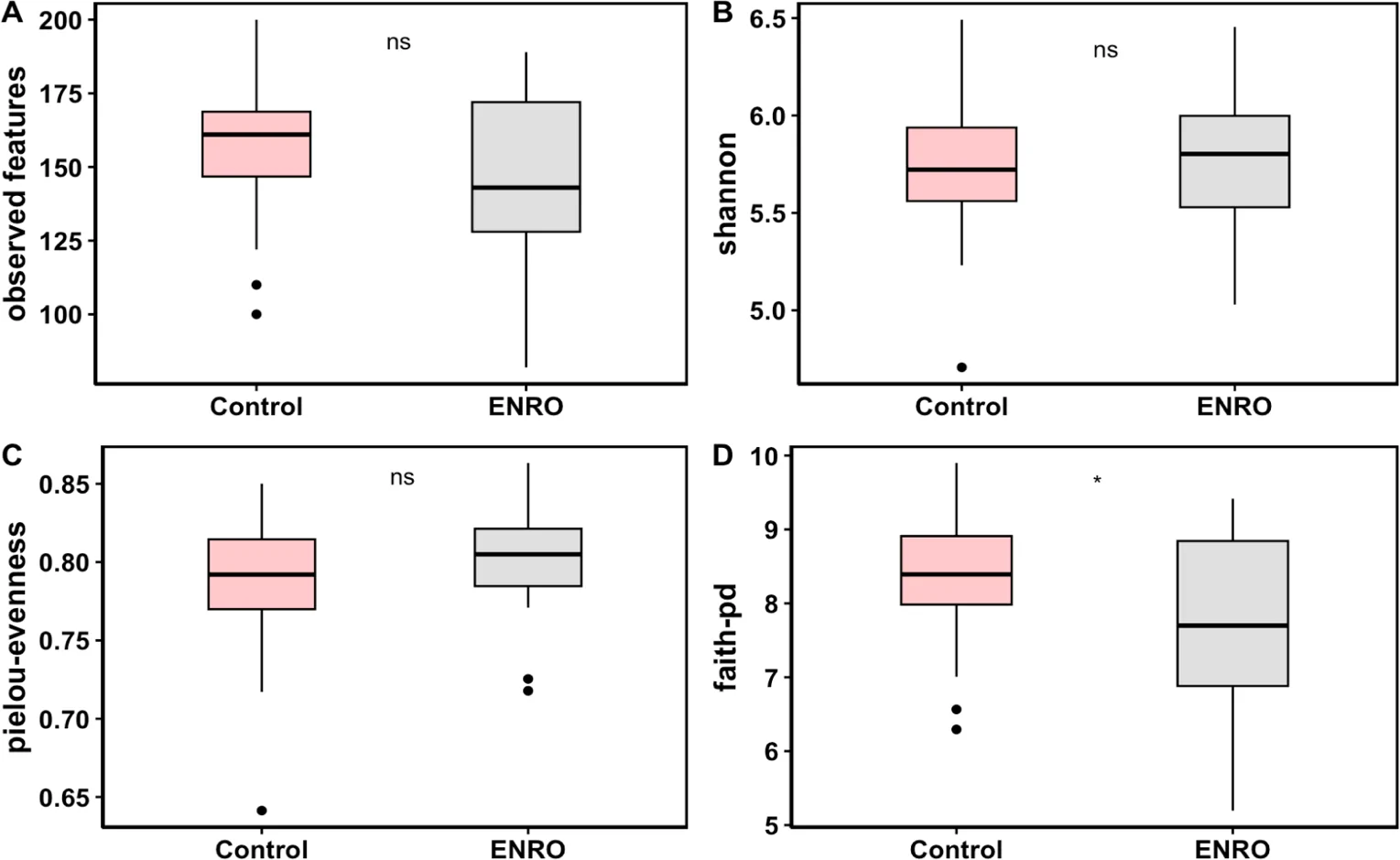
Boxplots for observed features (**A**), Shannon (**B**), Pielou’s evenness (**C**), and Faith’s pd (**D**) α-diversity metrics for the cecal microbiota of the ENRO treatment group and the control group. Each box shows the overall combined data from the entire experiment within each group. Horizontal bars inside boxes mark the midpoint of the data. The upper and lower vertical bars represent data values outside the middle 50%. The *P* values were calculated using a non-parametric Wilcoxon test (*P value < 0.05; ns, not significant).

**Fig 3:**
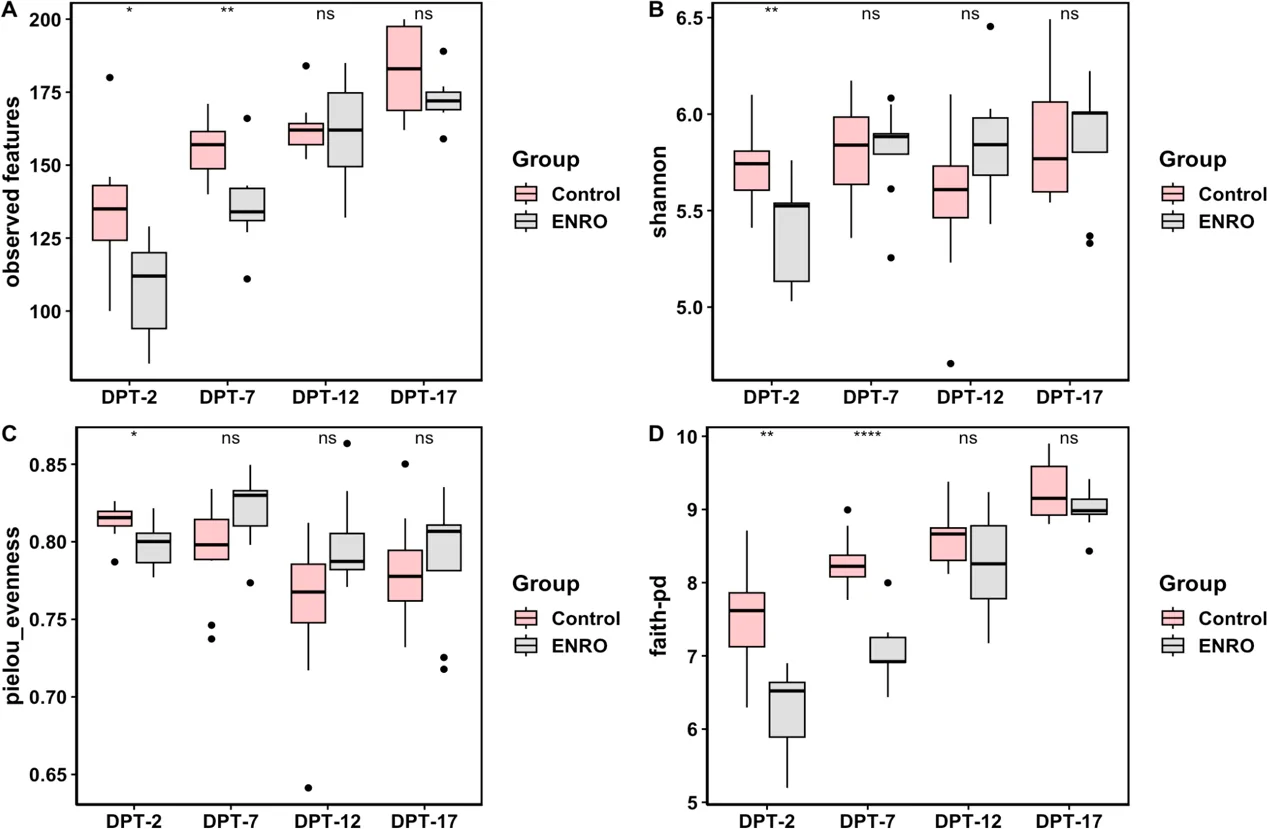
Boxplots for observed features (**A**), Shannon (**B**), Pielou’s evenness (**C**), and Faith’s pd (**D**) α-diversity metrics for the cecal microbiota of the ENRO treatment group and the control group on DPT-2, -7, -12, and -17. Each box shows the combined data from a particular sampling time point within each group. Horizontal bars inside boxes mark the midpoint of the data. The upper and lower vertical bars represent data values outside the middle 50%. The *P* values were calculated using a non-parametric Wilcoxon test (****P value < 0.0001, **P value < 0.01, *P value < 0.05; ns, not significant). DPT, day post the initiation of ENRO treatment.

Beta diversity analysis was measured by unweighted UniFrac distances (a measure of community dissimilarity that incorporates phylogenetic relationships between the features) and illustrated in a PCoA plot (Fig. 4). Two-dimensional visualizations of unweighted UniFrac distance (Fig. 4A) showed that the cecal microbiota of the ENRO treatment group was clustered closely and had a distinct separation from the microbiota cluster of the control group. Furthermore, samples from the same group and sampling time point formed relatively distinct clusters (Fig. 4B). ANOSIM was also conducted to further identify the dissimilarity source and confirm the results from the beta diversity analysis. ANOSIM test results indicated that the sources of dissimilarity of the cecal microbiomes between the two groups were ENRO treatment, the age of birds, and the interaction effect of antibiotic treatment with bird age (Fig. 5). ANOSIM tests further revealed that the dissimilarity in the cecal microbiota within the ENRO treatment group was considerably lower than that within the control group (Fig. 5A). Therefore, it is evident that ENRO treatment had a significant impact on the gut microbiota as shown by the PCoA analysis and the ANOSIM test. Beta diversity results revealed significant differences in the microbial structure between the groups; both antibiotic treatment and bird age contributed to the differences.

**Fig 4:**
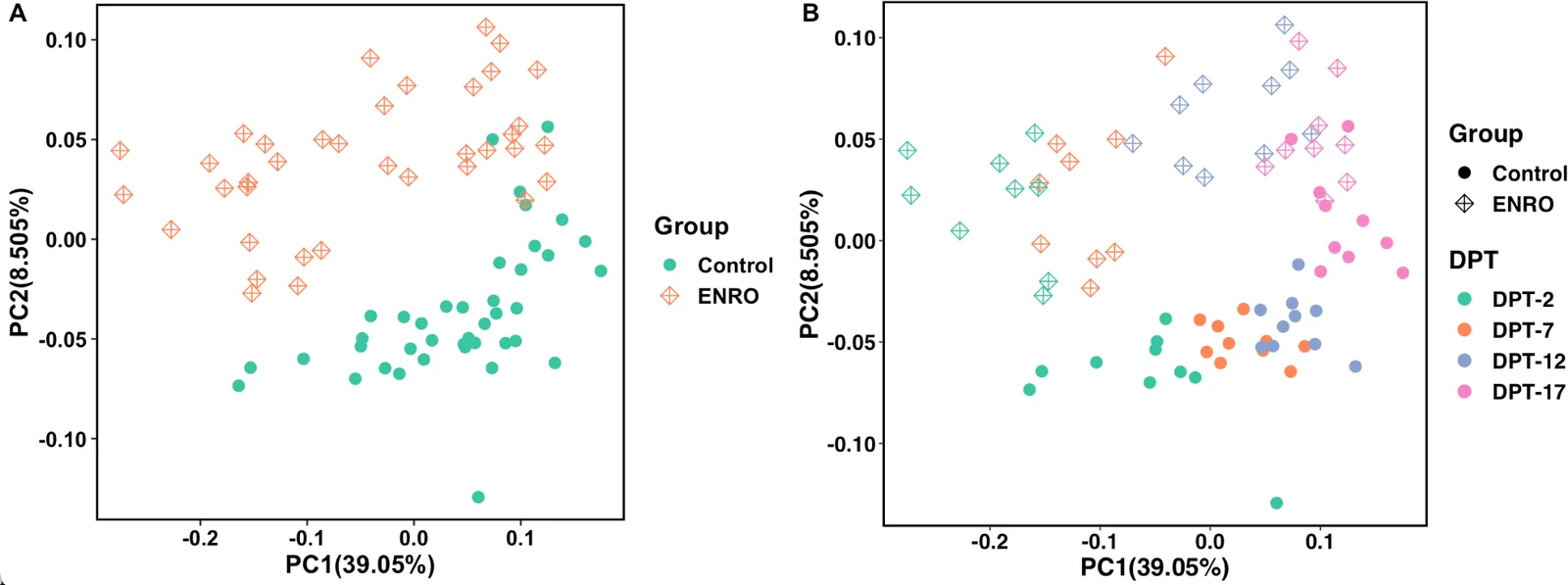
PCoA plots based on unweighted UniFrac distances for the ENRO treatment and the control groups analyzed by treatment status (**A**) or by treatment status × sampling time point (**B**). The PC1 axis depicts 39.05% of the total variance, and the PC2 axis shows 8.505% of the total variance. Each dot represents microbiota composition in a single sample. DPT, day post-initiation of ENRO treatment.

**Fig 5:**
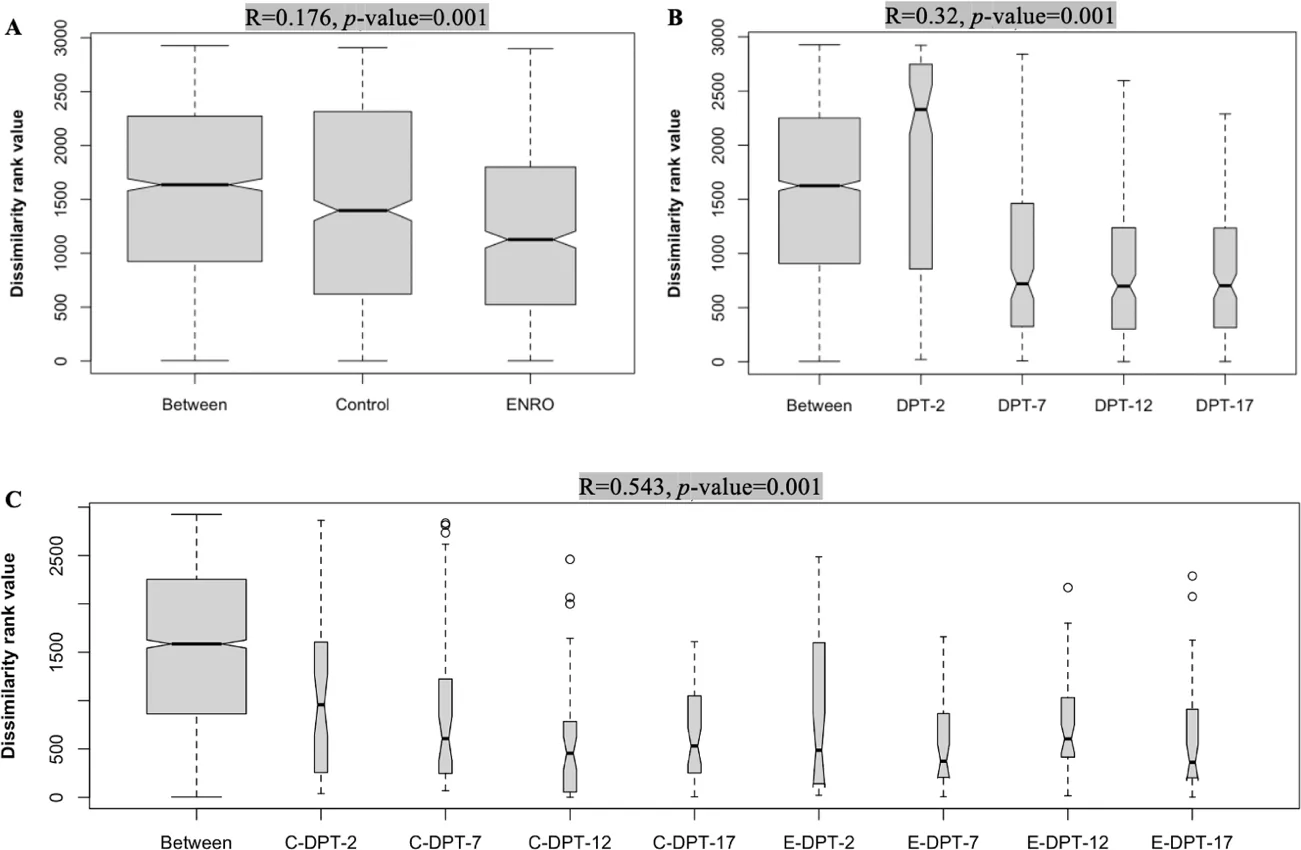
Dissimilarity in microbiota composition between and within groups (ENRO treatment and control) according to ANOSIM with the box plots showing the dissimilarity ranks for antibiotic treatment (**A**), chicken age (**B**), and the combined effect of antibiotic treatment and bird age. The analysis was conducted using a Bray-Curtis dissimilarity index based on the ASV composition of the total samples within each unit shown on the x-axis. The dissimilarity rank values calculated based on 999 permutations between and within groups are shown on the y-axis. In panel **A**, “Between” indicates the compositional dissimilarities between the ENRO treatment group and the control group; in panel **B**, “Between” indicates the compositional dissimilarities among all the sampling time points from both groups; in panel **C**, “Between” indicates the compositional dissimilarities among eight groups separated by treatment status (C, control; E, ENRO) and sampling time points. The test result is represented by an R value and the test significance value (*P* value). An R value close to “1” suggests strong dissimilarity between groups, while an R value close to “0” suggests less dissimilarity between groups. A *P* value less than 0.05 is generally considered to be statistically significant.

Taxonomic analysis was conducted using ASV data from 16S rRNA gene sequencing to determine the gut microbiota composition (Fig. 6). Four phyla (*Firmicutes*, *Proteobacteria*, *Actinobacteriota*, and *Campylobacterota*) were detected in all samples. The main phylum across the samples was *Firmicutes*, with a relative abundance of 95.71% and 93.32% in the ENRO treatment and the control groups, respectively. The three next most abundant phyla were *Campylobacterota* (2.56%), *Proteobacteria* (0.98%), and *Actinobacteriota* (0.74%) in the ENRO treatment group, while these were *Proteobacteria* (2.60%), *Actinobacteriota* (2.15%), and *Campylobacterota* (1.93%) in the control group (Fig. 6A). At the genus level, the three most abundant genera were *Ruminococcus_torques_group* (23.68%), *Lachnospiraceae (f)* (19.12%), and *Clostridia_vadinBB60_group* (9.45%) in the ENRO treatment group, and were *Lachnospiraceae(f)* (22.05%), *Ruminococcus_torques_group* (18.97%), and *Clostridia_vadinBB60_group* (14.16%) in the control group (Fig. 6B).

**Fig 6:**
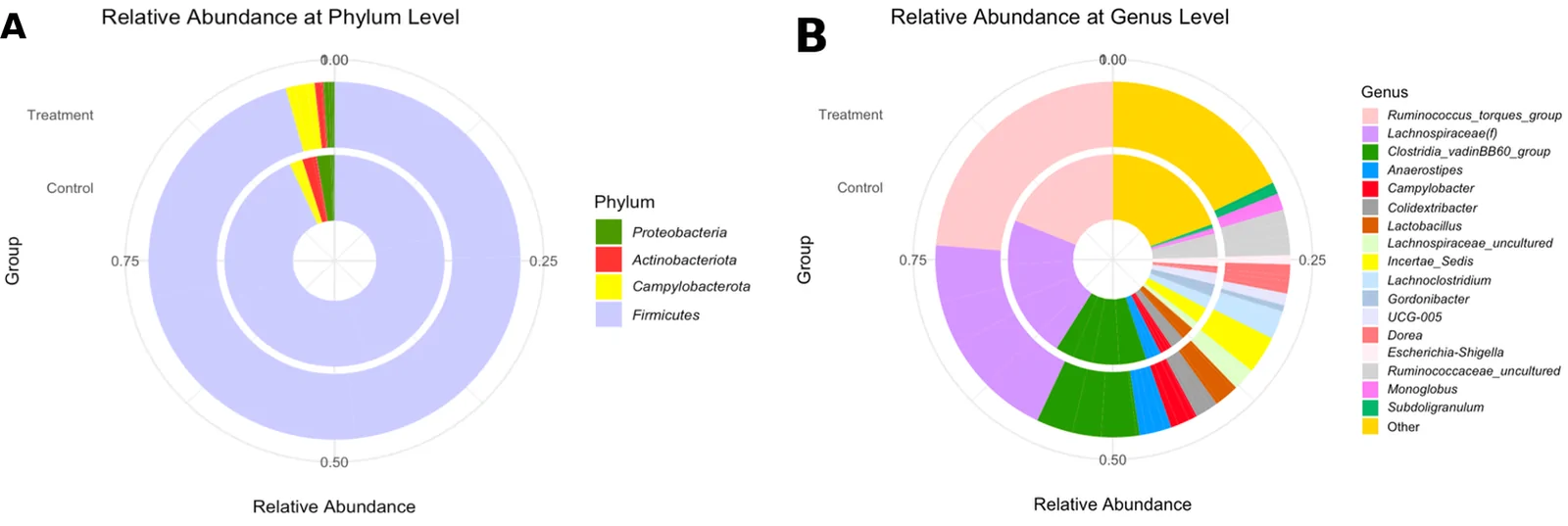
16S rRNA gene-based taxonomic composition of cecal microbiota at the phylum level (**A**) and the genus level (**B**) as shown in double doughnut plots. Outer ring: ENRO treatment group; inner ring: control group.

Even though the overall cecal microbiota compositions between the ENRO treatment group and the control group were highly similar (Fig. 6), significant differences were detected in the relative abundance of several taxa found in both groups as determined by the ANCOM-BC test (Fig. 7). At the phylum level, *Actinobacteriota* was significantly more abundant in the control group than the ENRO treatment group (Fig. 7A). At the genus level, *Proteus*, *Blautia*, *Romboutsia*, *Erysipelotrichaceae_uncultured*, *Christensenellaceae_R-7_group*, *RF39*, *Candidatus_Arthromitus*, *Weissella*, *Butyricicoccus*, and *Clostridia_vadinBB60_group* had a log-fold change (LFC) less than 0, indicating that these genera were significantly more abundant in the control group than the ENRO treatment group (Fig. 7B). In contrast, the abundance of the genera *Clostridium_innocuum_group* was significantly increased after the ENRO treatment (Fig. 7B).

**Fig 7:**
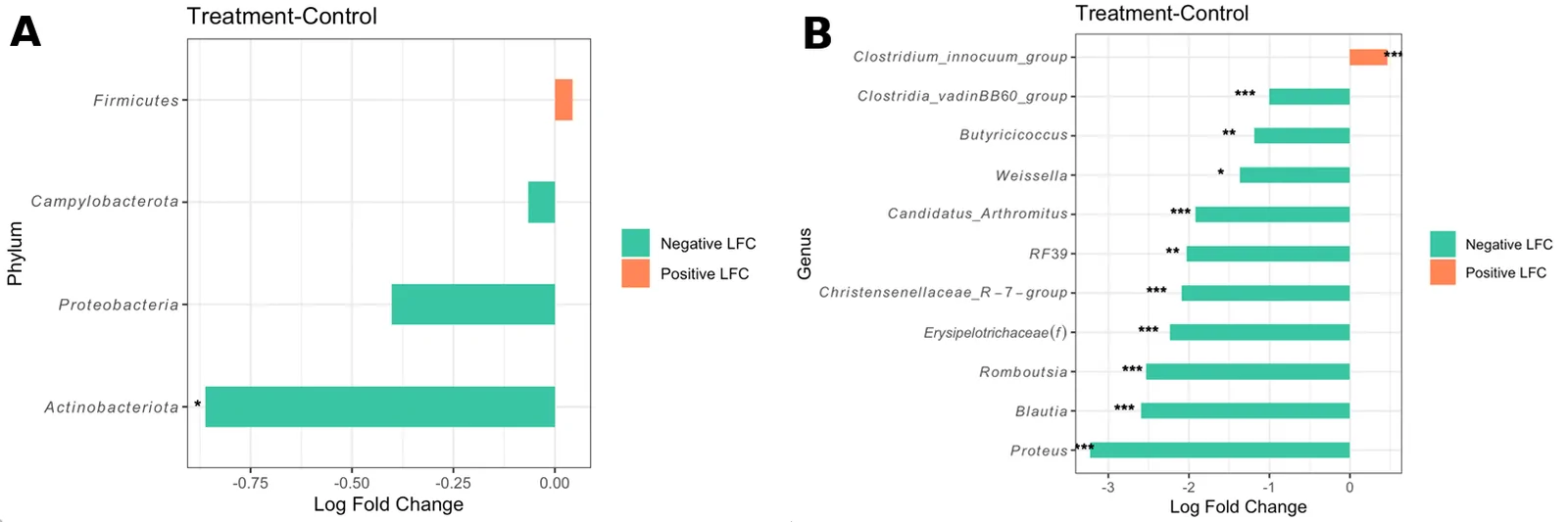
Bar plot of differentially abundant phyla (**A**) and genera (**B**) of the cecal microbiota obtained from ANCOM-BC analysis. Data are represented by LFC on the x-axis. A positive LFC (orange color) indicates that the corresponding taxon is more abundant in the ENRO treatment group compared with the control group. A negative LFC (green color) indicates that the corresponding taxon is more abundant in the control group than the ENRO treatment group. A significant LFC means the corresponding phylum or genus is significantly differentially abundant between the two groups. The significance level of LFC is shown by asterisks (***P value < 0.001, **P value < 0.01, and *P value < 0.05). RF39: *Bacilli*_RF39.

Pearson’s correlation analysis highlighted distinct correlations between *Campylobacter* abundance and other predominant members of the cecal microbiota. When total *Campylobacter* population (FQ-susceptible and FQ-resistant) was considered together, *Lactobacillus* showed a positive correlation (r = 0.36), whereas taxa, such as *Lachnospiraceae* _*uncultured* (r = −0.41) and *Incertae Sedis* (r = −0.34), were negatively correlated (Fig. 8). When only FQ-susceptible *Campylobacter* (control group) was considered, it had negative correlations with multiple genera, including *Clostridia vadinBB60 group* (r = −0.46), *Lachnospiraceae* _*uncultured* (r = −0.35), *Ruminococcaceae*_*uncultured* (r = −0.35), and *Gordonibacter* (r = −0.33). These findings indicate that *Campylobacter* abundance showed different correlation patterns with other cecal bacterial taxa depending on treatment status and FQ resistance phenotype (Fig. 8). Conversely, when only FQ-resistant *Campylobacter* (ENRO treatment group) was accounted for, a strong positive correlation with *Lactobacillus* (r = 0.48) and notable negative correlations with *Lachnospiraceae*_*uncultured* (r = −0.60) and several other taxa were observed (Fig. 8). These findings indicate that FQ-resistant and FQ-susceptible *Campylobacter* differ markedly in their microbial correlation networks, with ENRO treatment fostering certain microbiota members that may support proliferation of FQ-resistant *Campylobacter* while suppressing potentially antagonistic taxa.

**Fig 8:**
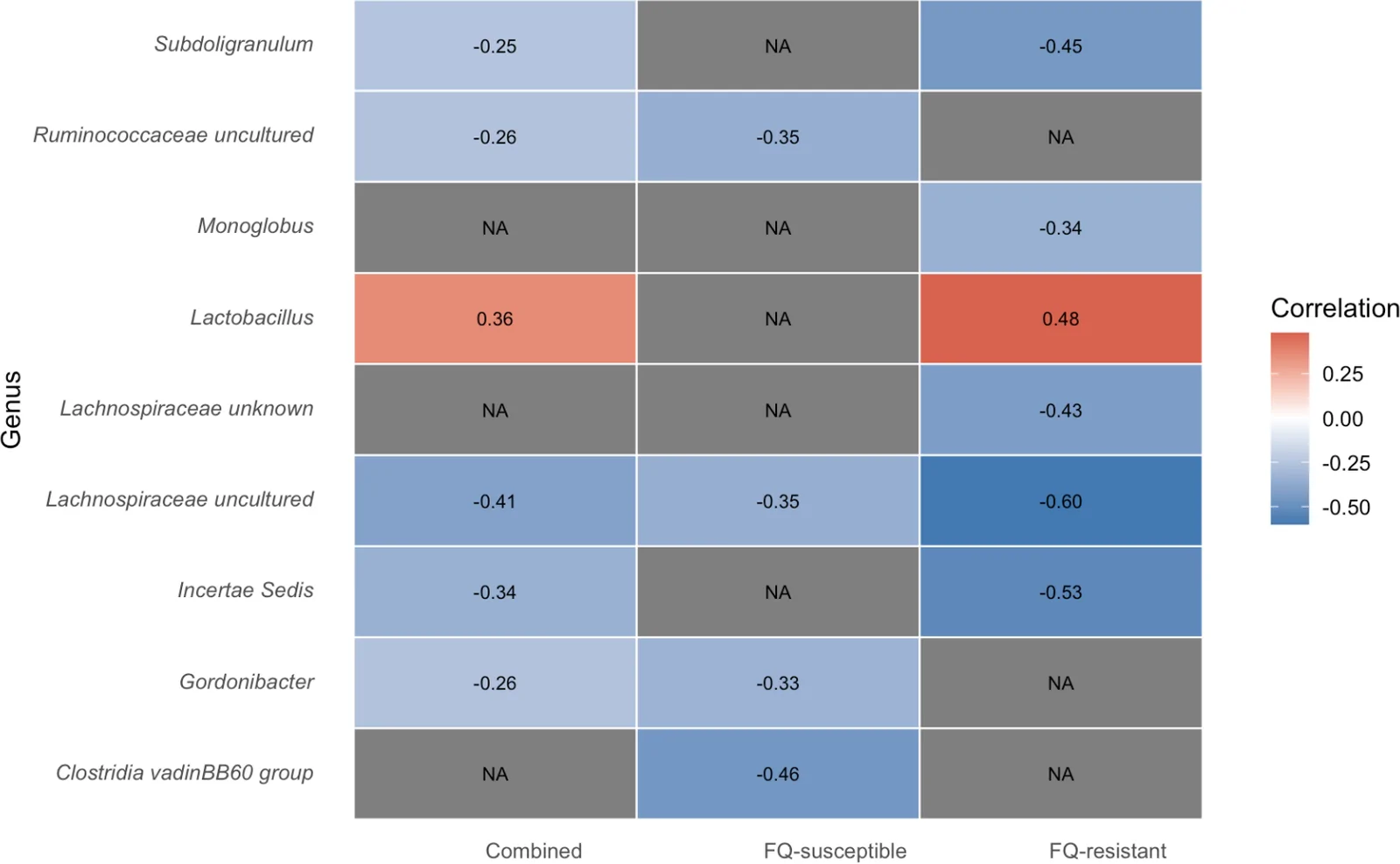
Heat map showing Pearson’s correlation coefficients for *Campylobacter* from both the ENRO-treated and control groups (combined) or separately (assuming *Campylobacter* from control group are FQ-susceptible and *Campylobacter* from ENRO-treated group are FQ-resistant) and other predominant genera at the genus level as determined by the 16S rRNA gene sequencing taxonomic composition analysis. Significant correlations are colored in red (positive) or blue (negative). Only statistically significant (*P* value < 0.05) correlation coefficients are shown. Coefficients range from −1 to 1, with 1 representing the highest positive correlation and −1 representing the highest negative correlation.

### Impact of ENRO treatment on the chicken cecal metabolite profiles

Untargeted metabolomics analysis identified a total of 1,038 metabolites in 89 cecal samples of the broiler chickens in three different groups: the baseline group (*n* = 9; non-colonized, non-treated), ENRO treatment group (*n* = 40; colonized with *C. jejuni*, ENRO-treated), and control group (*n* = 40; colonized with *C. jejuni*, non-treated). The total number of compounds with a known structural identity was 793, consisting of 261 lipids, 218 amino acids, 40 carbohydrates, 54 cofactors and vitamins, 11 metabolites related to energy production metabolism, 49 nucleotides and partially characterized nucleotides, 36 peptides, and 114 xenobiotics (Fig. 9).

**Fig 9:**
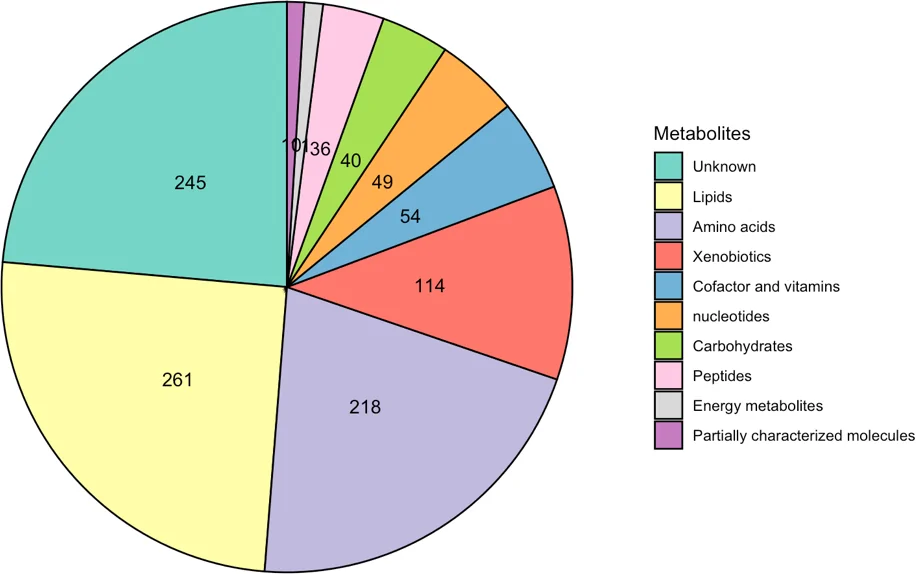
Pie chart depicting metabolic super pathways of the detected metabolites in the cecal contents of broiler chickens in this study, including all three groups (baseline, ENRO treatment, and control). Numbers of metabolites detected in each super pathway are shown in the corresponding category.

PCA was employed to highlight similarities and differences, and to discern patterns within the metabolomic data by the experimental groups. The baseline group (samples collected prior to *Campylobacter* inoculation and ENRO treatment) was clearly separated from the other two groups, while the overall separation of the control and ENRO treatment groups was minimal (Fig. 10A). However, when samples were grouped according to the sampling time point, clustering of data from the same group and sampling time point together was more pronounced (Fig. 10B). Based on the PCA results, further metabolite composition comparisons were focused mainly on the ENRO treatment and the control groups at different sampling time points, as detailed below.

**Fig 10:**
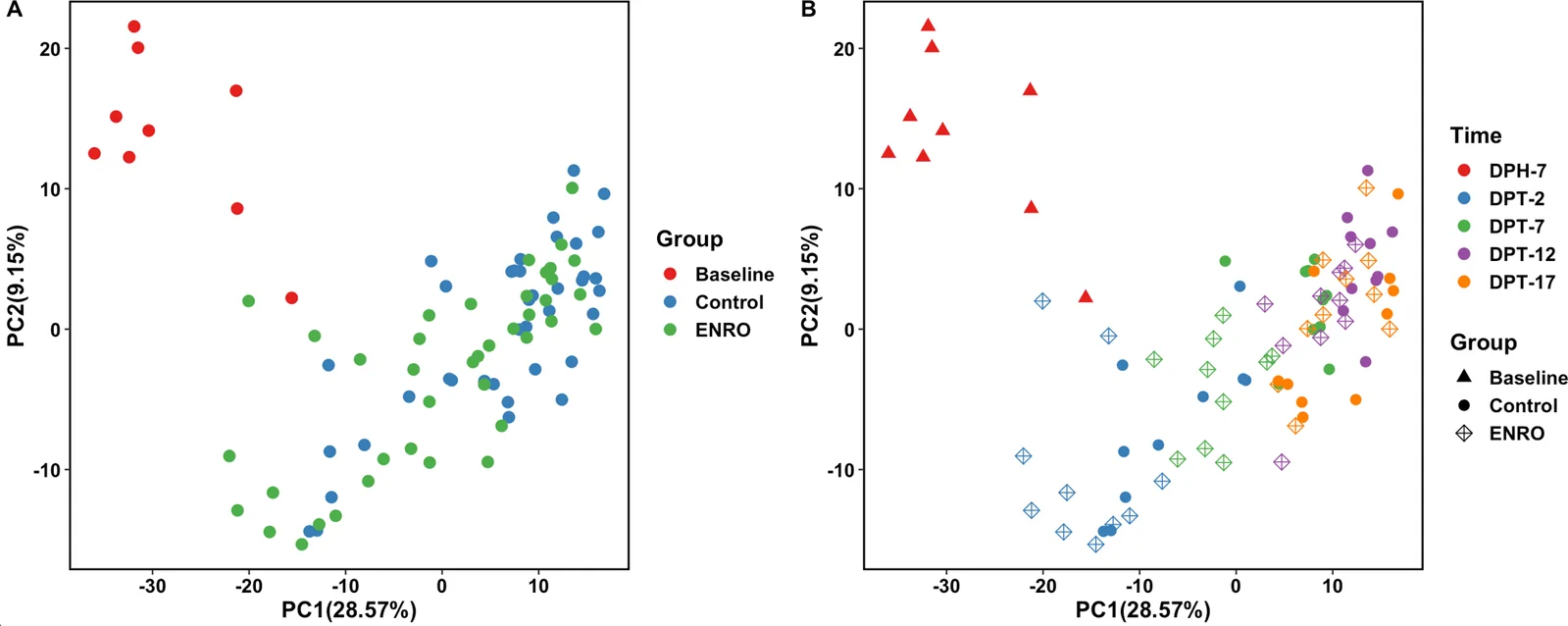
PCA plot based on intensity profiles of all annotated metabolites showing segregation of three treatment groups (**A**), and the segregation of groups stratified by sampling time points (**B**). The PC1 axis depicts 28.57% of the total variance, and the PC2 axis shows 9.15% of the total variance. Each dot represents metabolite composition in a single cecal sample. DPH, day post-hatch (non-colonized, non-treated baseline group). DPT, day post-initiation of ENRO treatment.

For the 793 metabolites with known structures, we compared the fold changes in metabolite abundances between the ENRO treatment group and the control group at 4 different sampling time points and across all times combined. Sixty-four compounds were considered as differentially abundant metabolites after the ENRO treatment, according to Welch’s two-sample *t*-test (Table 2). Among these, 51 were upregulated and 13 were downregulated in the ENRO treatment group compared with the control group, based on the predefined conservative criteria of fold-change >2 for upregulation, fold-change <0.5 for downregulation, and *q* value <0.05. The identified metabolites and their associated super pathways, the fold changes at different sampling times, and the *q* values are depicted in Table 2.

**TABLE 2:** Significantly altered metabolites (*n* = 64) in the ceca of combined ENRO treatment and control groups, as determined by Welch’s two-sample *t*-test and fold-change heatmap^*a*^^,^^*b*^^,^^*c*^^,*d*,^^*e*^

No.	Biochemical name	E-DPT-2/C-DPT-2		E-DPT-7/ C-DPT-7		E-DPT-12/C-DPT-12		E-DPT-17/C-DPT-17		ENRO/Control	
		Fold change	*q* value	Fold change	*q* value	Fold change	*q* value	Fold change	*q* value	Fold change	*q* value
Amino acid											
1	N-methylglutamate	0.34	0.528	0.28^˅^	0.001	0.34	0.543	0.11^˅^	0.002	0.21^˅^	0.000
2	Phenethylamine	0.86^˅^	0.140	14.92^˄^	0.003	240.47^˄^	0.096	186.86^˄^	0.007	115.38^˄^	0.000
3	3-Methylglutaconate	0.6^˅˅^	0.234	2.45^˄^	0.000	8.84	0.415	6.59^˄^	0.002	2.36^˄^	0.000
4	Creatinine	0.76^˅^	0.000	89.12^˄^	0.000	2.52^˄^	0.000	1.24^˄^	0.027	1.79^˄^	0.013
5	2-Methylserine	1.43^˄^	0.119	3.7^˄^	0	2.76^˄^	0	1.05	0.075	2.08^˄^	0
6	3-Methylhistidine	1.41^˄^	0.105	3.53^˄^	0.008	2.55^˄^	0.004	1.02^˄^	0.008	2.02^˄^	0.004
7	1-Methyl-5-imidazoleacetate	0.02^˅^	0.1	0.03^˅^	0.0006	0.46^˅^	0.006	0.47^˅^	0.0025	0.29^˅^	0
8	N-acetyl-3-methylhistidine*	1.3^˄^	0.105	5.13	0.132	4.06^˄^	0.093	3.44	0.05	2.77^˄^	0
9	N6,N6,N6-trimethyllysine	1.44	0.265	2.23^˄^	0.025	14.98^˄˄^	0.129	3.98^˄^	0.021	2.5^˄^	0
10	Hydroxy-N6,N6,N6-trimethyllysine*	1.88^˄˄^	0.234	2.11	0.069	4.42	0.5	1.55	0.081	2.28^˄^	0.003
11	N,N,N-trimethyl-5-aminovalerate	0.69	0.225	0.54	0.085	0.38^˅^	0.095	0.37	0.124	0.47^˅^	0
12	N-acetylmethionine sulfoxide	8.2	0.395	1.28^˄^	0	2.53	0.544	1.71^˄^	0.001	3.21^˄^	0
13	4-Methylthio-2-oxobutanoate	0.1	0.358	0.28^˅^	0.016	0.26	0.298	0.98	0.041	0.34^˅^	0.002
14	Carboxymethylarginine	1.24^˄^	0.001	3.21^˄^	0.001	2.42^˄^	0.009	2.93^˄^	0.006	1.8^˄^	0.027
15	Creatine	33.18	0.565	2.31^˄^	0	1.47	0.486	1.03^˄^	0	4.97^˄^	0
16	Cyclo(his-pro) [peptide]	4.02	0.421	1.2^˄^	0	4.83^˄˄^	0.125	1.33^˄^	0.002	2.65^˄^	0.004
Lipid											
1	1-Stearoyl-2-linoleoyl-GPI (18:0/18:2)	3.70^˄^	0.156	2.85	0.161	2.52	0.267	5.53	0.186	3.58^˄^	0.000
2	2-Stearoyl-GPE (18:0)*	1.27	0.282	2.23^˄˄^	0.055	1.58	0.458	6.13^˄^	0.015	2.39^˄^	0.001
3	1-Oleoyl-GPI (18:1)	1.23	0.471	2.78^˄˄^	0.049	2.75	0.544	3.13^˄^	0.005	2.55^˄^	0.000
4	Butyrylcarnitine (C4)	1.4^˄˄^	0.178	1.5^˄^	0.014	3.82	0.242	4.92	0.05	2.64^˄^	0
5	5-Dodecenoylcarnitine (C12:1)	1.33	0.6	1.64	0.083	2.25^˄^	0.036	3.36^˄˄^	0.047	1.99^˄^	0
6	Deoxycarnitine	0.64	0.569	0.85	0.079	0.39	0.321	0.31	0.173	0.45^˅^	0
7	Choline phosphate	2.14	0.303	3.07	0.075	7.38	0.237	194.13	0.186	5.46^˄^	0
8	1-Palmitoyl-2-linoleoyl-galactosylglycerol (16:0/18:2)*	0.36^˅^	0.086	0.31^˅˅^	0.055	0.50	0.171	0.34	0.114	0.39^˅^	0.000
9	Glycerophosphorylcholine (GPC)	2.96	0.663	8.14	0.26	2.21	0.269	10.85	0.083	4.02^˄^	0
10	1-Stearoyl-2-oleoyl-GPC (18:0/18:1)	0.92	0.44	2.95	0.334	2.84	0.29	3.5	0.086	2.22^˄^	0.028
11	1-Stearoyl-2-arachidonoyl-GPE (18:0/20:4)	1.49	0.505	2.56	0.256	2.14	0.459	2.54	0.123	2.13^˄^	0.008
12	1-Stearoyl-2-oleoyl-GPI (18:0/18:1)*	1.61	0.441	1.85	0.162	2.71	0.377	3.88	0.2	2.57^˄^	0.003
13	1-Stearoyl-2-linoleoyl-GPC (18:0/18:2)*	0.97	0.521	2.71	0.327	1.97	0.294	3.85	0.077	2.05^˄^	0.010
14	1-Palmitoyl-GPC (16:0)	2.13	0.279	2.06	0.311	1.82	0.539	2.37	0.192	2.07^˄^	0
15	1-Stearoyl-GPC (18:0)	1.78	0.323	2.12	0.229	1.92	0.548	3.25	0.146	2.19^˄^	0.001
16	1-Stearoyl-GPE (18:0)	1.77	0.251	2.57	0.073	1.8	0.415	3.9	0.083	2.4^˄^	0.001
17	1-Linoleoyl-GPE (18:2)*	2.37	0.551	1.67^˄˄^	0.041	2.3	0.325	2.47	0.072	2.17^˄^	0.002
18	1-Oleoylglycerol (18:1)	2.48	0.381	1.67	0.338	1.66	0.448	2.21	0.128	1.97^˄^	0.001
19	1-Linoleoylglycerol (18:2)	2.14	0.483	2.18	0.314	1.49	0.467	2.22	0.116	1.97^˄^	0.001
20	1-Dihomo-linolenylglycerol (20:3)	2.96	0.43	1.88	0.207	1.29	0.271	2.65^˄^	0.028	2.14^˄^	0.003
21	1-Arachidonylglycerol (20:4)	3.75	0.555	3.82	0.331	2.45	0.436	4.59	0.123	3.67^˄^	0
22	Stearoyl-linoleoyl-glycerol (18:0/18:2) [2]*	2.61^˄^	0.019	1.14	0.279	2.51^˄^	0.103	2.57^˄^	0.007	2.29^˄^	0.002
23	Oleoyl-linoleoyl-glycerol (18:1/18:2) [2]	3.81^˄˄^	0.238	1.84	0.278	2.15	0.236	1.98	0.094	2.28^˄^	0
24	Galactosylglycerol	0.23	0.627	0.51	0.189	0.54	0.47	0.87	0.171	0.45^˅^	0.02
25	Ceramide (d18:1/17:0, d17:1/18:0)*	1.69^˄^	0.151	2.19^˄˄^	0.041	2.1	0.175	3.96	0.171	2.48^˄^	0
26	N-stearoyl-sphingosine (d18:1/18:0)*	1.51	0.343	2.01	0.075	2.07	0.286	3.71	0.171	2.26^˄^	0
27	Stearoyl sphingomyelin (d18:1/18:0)	1.34	0.464	2.85^˄˄^	0.059	3.15	0.443	6.06	0.094	3.13^˄^	0
28	Sphingomyelin (d18:1/17:0, d17:1/18:0, d19:1/16:0)	1.34	0.347	2.4	0.065	3.2	0.438	5.7	0.074	3.07^˄^	0
Carbohydrate											
1	Arabonate/xylonate	3.10^˄^	0.089	3.92^˄^	0.000	1.38^˄^	0.041	0.67	0.074	2.16^˄^	0.006
2	Stachyose	0.90^˅^	0.126	0.05^˅˅^	0.037	1.05^˄˄^	0.149	0.17^˅^	0.008	0.33^˅^	0.033
3	Trehalose	0.12	0.246	0.56^˅˅^	0.049	0.74^˅^	0.003	0.84	0.114	0.28^˅^	0.003
Xenobiotics											
1	Catechol sulfate	0.47	0.499	5.37	0.306	3.19^˄^	0.045	1.26^˄^	0.018	2.02^˄^	0.036
2	1-Methylxanthine	0.99^˅^	0.010	19.38^˄^	0.000	11.65^˄^	0.000	4.52	0.227	2.11^˄^	0.004
3	Dihydrocaffeate sulfate (2)	19.61	0.476	4.68^˄^	0.000	1.46^˄˄^	0.154	0.49^˅^	0.003	8.09^˄^	0.038
4	Deoxymugineic acid	1.06^˄^	0.000	261.68^˄^	0.000	23.56^˄^	0.000	0.59^˅^	0.001	2.42	0.036
5	N1,N10-dicoumaroylspermidine	49.00^˄^	0.000	0.52^˅^	0.001	0.80^˅^	0.000	0.84^˅^	0.000	8.49^˄^	0.050
6	Dihydroferulate	0.45^˅^	0.008	0.20^˅^	0.000	0.66^˅^	0.000	0.50	0.117	0.46^˅^	0.000
7	Nicotianamine	1.15^˄^	0.000	15.27^˄^	0.000	2.45^˄^	0.000	0.89^˅^	0.008	1.97^˄^	0.033
8	2-Dimethylaminoethanol	0.28	0.603	1.47	0.002	1.15	0.237	0.10^˅^	0.005	0.35^˅^	0.051
9	Gluconate	5.84^˄^	0.141	1.76^˄^	0	1.68	0.176	0.93^˅^	0.001	3.49^˄^	0.032
10	6-Hydroxydaidzein	0.3	0.499	0.25	0.083	0.22	0.17	0.29	0.166	0.26^˅^	0
11	Feruloylquinate (4)	4.39	0.395	2.91^˄^	0	2.13^˄^	0.011	0.46^˅^	0.009	2.85^˄^	0.036
12	Feruloylputrescine	7.79^˄˄^	0.216	1.26^˄^	0.001	1^˄˄^	0.146	1^˄^	0.002	2.96^˄^	0.011
13	N-acetylpyrraline	0.17	0.464	0.91	0.166	1.77	0.417	0.26	0.195	0.45^˅^	0.041
14	Sulfociprofloxacin*	6.02^˄^	0.016	1.92^˄^	0.000	1.00^˄^	0.010	1.02^˄^	0.000	2.45^˄^	0.000

^*a*^ Symbol ^˄^ at the end of a numeric number indicates the metabolite is significantly upregulated (fold-change > 2; *q* value < 0.05).

^*b*^ Symbol ^˅^ indicates the metabolite is significantly downregulated (fold-change < 0.5; *q* value < 0.05) in the ENRO group compared with the control group shown in the top row.

^*c*^ Symbols ^˄˄^ and ^˅˅^ indicate the metabolite is upregulated or downregulated, respectively, with a narrowly missed statistical cutoff for significance (0.05 < *q* value < 0.10). *q* values for each comparison are derived from the natural log-transformed data using the false discovery rate method; the *q* value is the expected proportion of false positives among results deemed significant.

^*d*^ The “*” sign at the end of a molecule denotes isotope labeling of the carbon atom.

^*e*^ Fold-change describes the ratio of the mean scaled intensity for a metabolite between two experimental groups (E, ENRO treatment group; C, control group; DPT, day post-initiation of ENRO treatment).

Lipid metabolism was the most affected pathway at the super pathway level, given that it had the greatest number of significantly altered metabolites (28/64) (Table 2). Amino acid metabolism was the second most affected pathway (15/64 metabolites) and had the overall most affected metabolite, phenethylamine, with the greatest fold change observed among all the significantly altered metabolites. Xenobiotic metabolism was the third most affected pathway, representing 21.88% (14/64) of the altered metabolites. Other significantly altered metabolites were from carbohydrate (3/64), cofactors and vitamins (3/64), and peptide (1/64) super pathways (Table 2). The altered lipid metabolites were categorized into nine sub-pathways, with phospholipid metabolism as the most affected sub-pathway based on the fold-change values of the metabolites. Choline phosphate (fold-change = 5.46) and glycerophosphorylcholine (fold-change = 4.02) were the main altered metabolites in the phospholipid metabolism sub-pathway, and both were significantly upregulated after ENRO treatment. Galactosylglycerol (fold-change = 0.45) and 1-palmitoyl-2-linoleoyl-galactosylglycerol (16:0/18:2)* (fold-change = 0.39) were downregulated after ENRO treatment (both belong to the galactosyl glycerolipids metabolism sub-pathway). Deoxycarnitine (fold-change = 0.45) from the carnitine metabolism sub-pathway was also downregulated after ENRO treatment.

For the 15 metabolites altered in the amino acid metabolism super pathway, 11 were upregulated, and 4 were downregulated in the ENRO treatment group compared to the control group at any given or combined sampling time points (Table 2). Phenethylamine, a primary amine of the phenylalanine metabolism sub-pathway, overall drastically increased in abundance after ENRO treatment (except for DPT-2). More specifically, even though it was slightly downregulated during the beginning of the ENRO treatment period (fold-change = 0.86 at DPT-2), phenethylamine was upregulated remarkably (fold-changes = 14.92, 240.47, 186.86 at DPT-7, -12, -17, respectively) after the termination of ENRO treatment (DPT-5 was the last day of ENRO treatment).

Xenobiotics are chemical substances discovered within an organism that are not inherently produced or anticipated to be present in that organism (55). In this study, 14 compounds belonging to xenobiotics were found to be differentially abundant, with 10 upregulated and four downregulated in the ENRO treatment group compared with the control group (Table 2). It is worth mentioning that one such compound, sulfociprofloxacin (a metabolic product of enrofloxacin) from the antibiotic sub-pathway, was significantly upregulated during the ENRO treatment (fold-change = 6.02 at DPT-2), and the upregulation trend was observed until the end of the study.

### Correlation analysis of cecal microbiota and metabolites

To assess the overall association between the cecal microbiota composition (based on 16S rRNA gene sequence analysis) and metabolomic profiles, we performed Pearson’s correlation analysis with the relative abundance of the top 18 most abundant bacterial genera and all 64 differentially abundant metabolites intensity profiles (Fig. 11). When both FQ-susceptible and FQ-resistant *Campylobacter* populations (control and ENRO treatment groups) were taken into consideration, *Campylobacter* abundance was found to be significantly positively correlated with only one lipid metabolite, butyrylcarnitine (fold-change = 1.5 at DPT-7); however, it was significantly negatively correlated with 12 metabolites from across all the main super pathways. Interestingly, overall, the lipid super pathway generally had fewer significant correlations with the dominant cecal microbiota genera compared with other metabolic super pathways (Fig. 11). Of note, the most significantly altered metabolite, phenethylamine, did not correlate significantly with any of the top 18 most abundant bacterial genera of the cecal microbiota (Fig. 11).

**Fig 11:**
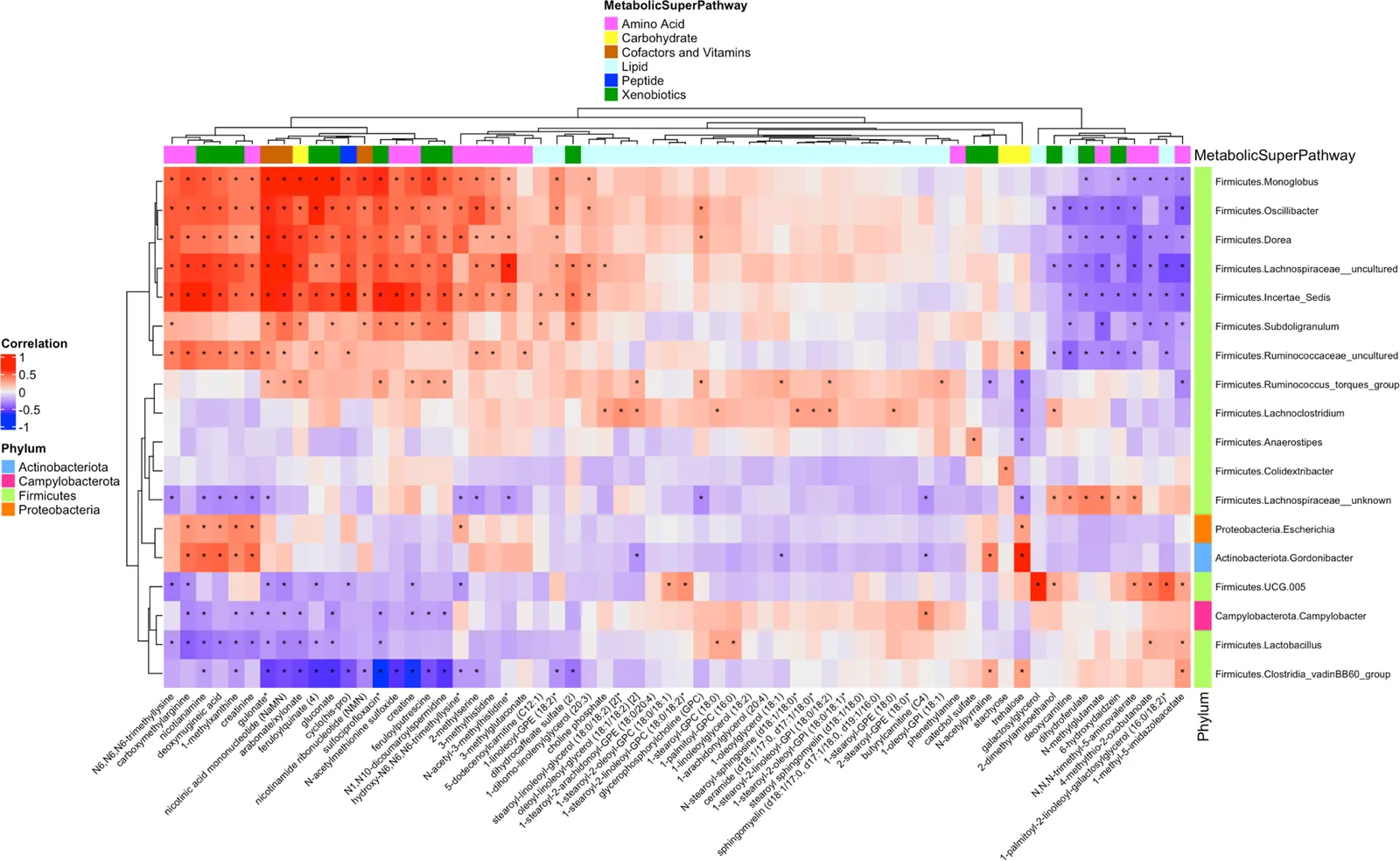
Heatmap depicting the correlations between the top 18 most abundant bacterial genera (based on 16S rRNA gene sequencing) (right y-axis) and all 64 differentially abundant metabolites (lower x-axis) in the combined control and ENRO treatment groups. Correlations are colored in red (positive) or blue (negative), and significant correlations (*P* value < 0.05) are marked with a *. Pearson’s coefficients range from −1 to 1, with 1 representing the highest positive correlation and −1 representing the highest negative correlation.

As can be seen in Fig. 12, metabolite*—Campylobacter* abundance relationships differed significantly by the FQ phenotype of the organism. Within the control group (FQ-susceptible *C. jejuni*), significant associations were much fewer and primarily negative, with only one positive association (1-palmitoyl-2-linoleoyl-galactosylglycerol). In contrast, the ENRO-treated group (FQ-resistant *C. jejuni*) exhibited a broader suite of negative correlations spanning cofactors/vitamins, xenobiotics, amino acids, and carbohydrates, with the most negatively correlated metabolite being sulfociprofloxacin. FQ-resistant *C. jejuni* had notable positive correlations with several metabolites; the strongest was with the histidine metabolite 1-methyl-5-imidazoleacetate (Fig. 12). Collectively, these data suggest that the relative abundance of FQ-resistant *C. jejuni* is linked to depletion of a broad set of metabolites along with a selective enrichment of a smaller group of amine and lipid derivatives.

**Fig 12:**
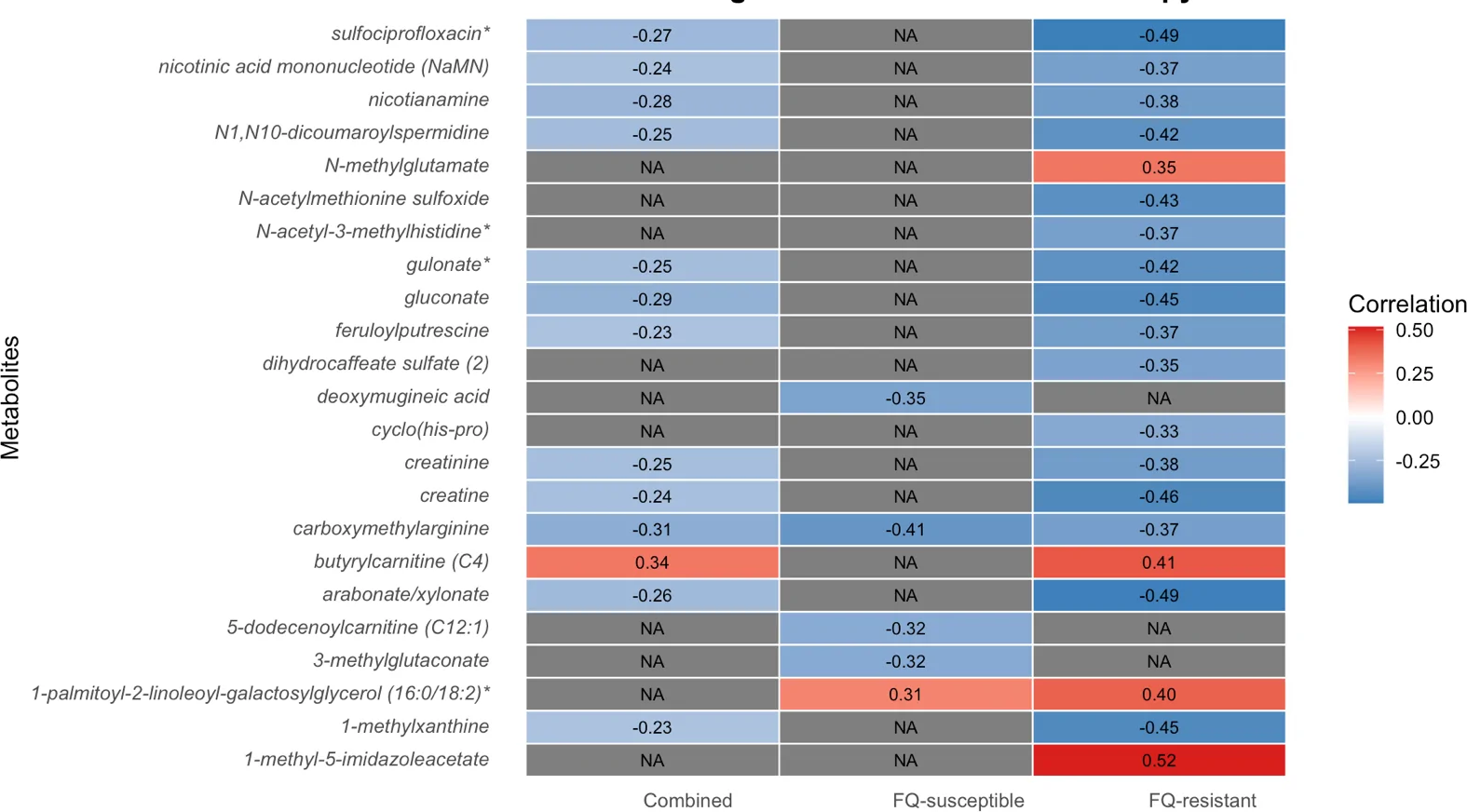
Heat map showing Pearson’s correlation coefficients for *Campylobacter* abundance from both the ENRO treatment and control groups (combined) or separately (FQ-susceptible and FQ-resistant) and significant differentially abundant metabolites. Significant correlations are colored in red (positive) or blue (negative). Only statistically significant (*P* value < 0.05) correlation coefficients are shown. Coefficients range from −1 to 1, with 1 representing the highest positive correlation and −1 representing the highest negative correlation.

### Shotgun metagenomics analysis

#### Top 10 most abundant bacterial species in cecal microbiota across treatment groups and time points

Since we did not observe any significant correlation between the cecal microbiota at the genus level (the highest taxonomic resolution achieved by the 16S rRNA gene sequencing analysis) and the differentially abundant metabolites, we proceeded with shotgun metagenomic sequencing to examine the gut microbial community at the species level and to assess the correlation between certain metabolites (e.g., phenethylamine) and specific bacterial species. To determine the dominant bacterial taxa in the chicken cecum across the study period, we analyzed shotgun metagenomic data to determine species-level relative abundances in each of the eight groups separated by treatment status (control and ENRO treatment) and sampling time points (day post-initiation of ENRO treatment, DPT-2, -7, -12, -17). For each group, the top 10 most abundant bacterial species were identified based on mean relative abundance, and only these were visualized (Fig. 13). Several taxa showed consistently high abundance across multiple sampling time points, including *Flavonifractor plautii*, *Dysosmobacter welbionis*, and *Lachnoclostridium phocaeense*. In particular, *Gordonibacter pamelaeae* dominated the control samples during the first sampling time (C-DPT-2) with over 13% mean relative abundance. Notably, the microbial community differed remarkably in both diversity and dominant species composition between the control and ENRO-treated groups on DPT-2, with the later time points (DPT-12 and DPT-17) showing more convergence of microbial profiles between the two groups (Fig. 13), possibly due to the decrease in antibiotic-driven selection pressure.

**Fig 13:**
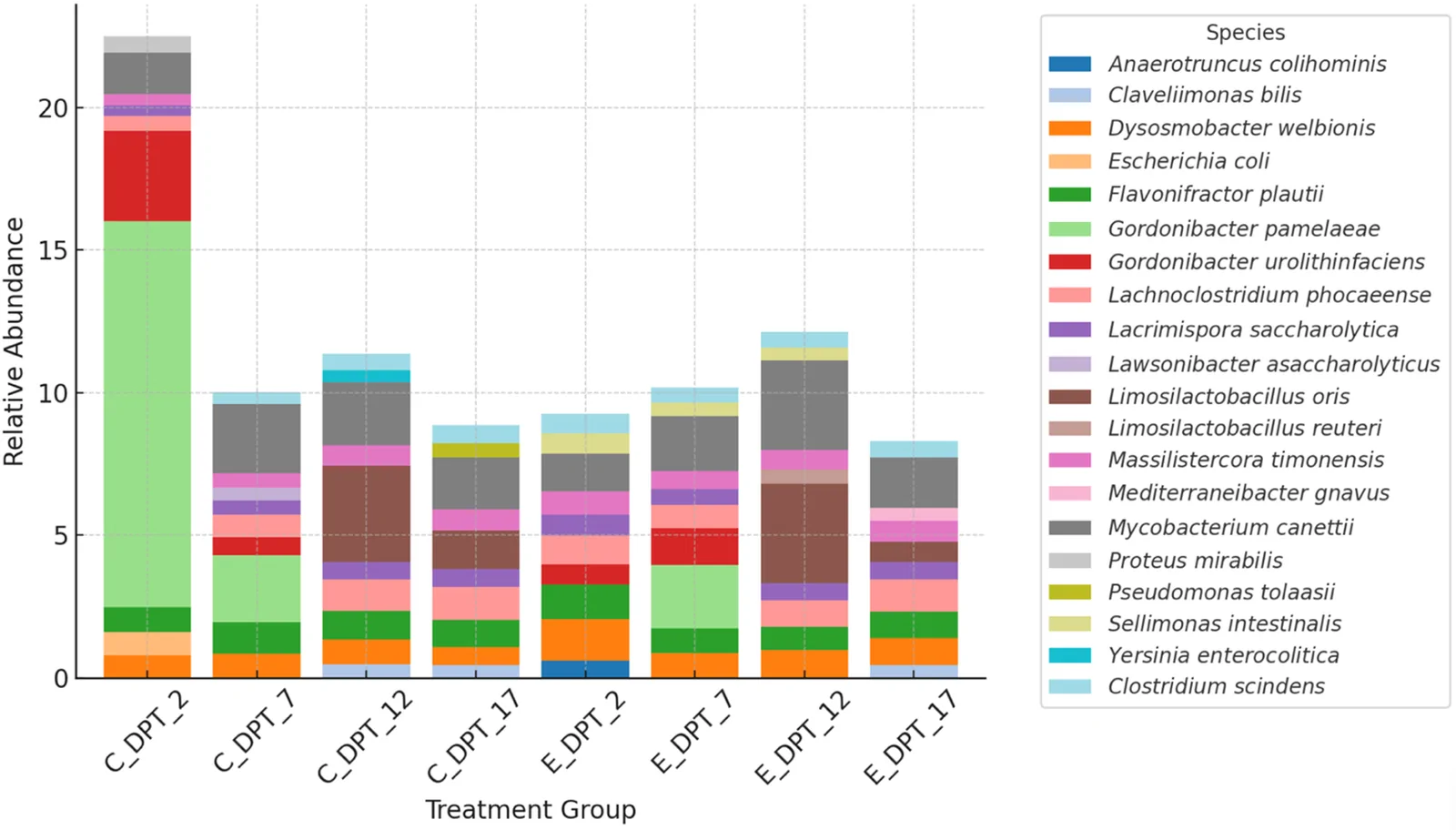
Stacked bar-plot representations of the main taxa’s relative abundances at the species level based on shotgun metagenomics analysis of the cecal microbiome. Reads assigned to *Homo sapiens*, “unclassified,” or “cannot be assigned to a (non-viral) species” were excluded to focus on the informative bacterial taxa. For each group (x-axis), only the top 10 most abundant bacterial species (based on mean relative abundance) were identified and visualized. C, control; E, ENRO treatment; DPT, day post-initiation of ENRO treatment.

#### Cecal microbiota members correlated with FQ-susceptible *C. jejuni*

To explore potential interactions between the cecal microbiota and FQ-susceptible *C. jejuni*, we calculated pairwise Pearson’s correlation coefficients between the abundances of *C. jejuni* and other microbial species in the cecal contents of birds in the control group (colonized with FQ-susceptible *C. jejuni* but not treated with ENRO) using the shotgun metagenomics data from across all sampling time points. Among the microbiota taxa, several species demonstrated strong positive or negative correlations with FQ-susceptible *C. jejuni*. Of the top 10 positively correlated species (Fig. 14), *Paenibacillus lutimineralis* (correlation coefficient = 0.765), *Mycoplasma wenyonii* (correlation coefficient = 0.704), and *Candidatus Nitrosotalea okcheonensis* (correlation coefficient = 0.703) had the highest correlation values, suggesting potential ecological compatibility or co-occurrence patterns with *C. jejuni* colonization in untreated birds. Conversely, of the most negatively correlated microbiota members (Fig. 14), *Occultella kanbiaonis* (correlation coefficient = −0.553), *Hydrogenimonas cancrithermarum* (correlation coefficient = −0.538), and *Erythrobacter aureus* (correlation coefficient = −0.536) were the top three taxa, implying possible microbial competition or exclusion dynamics within the gut environment of the control birds.

**Fig 14:**
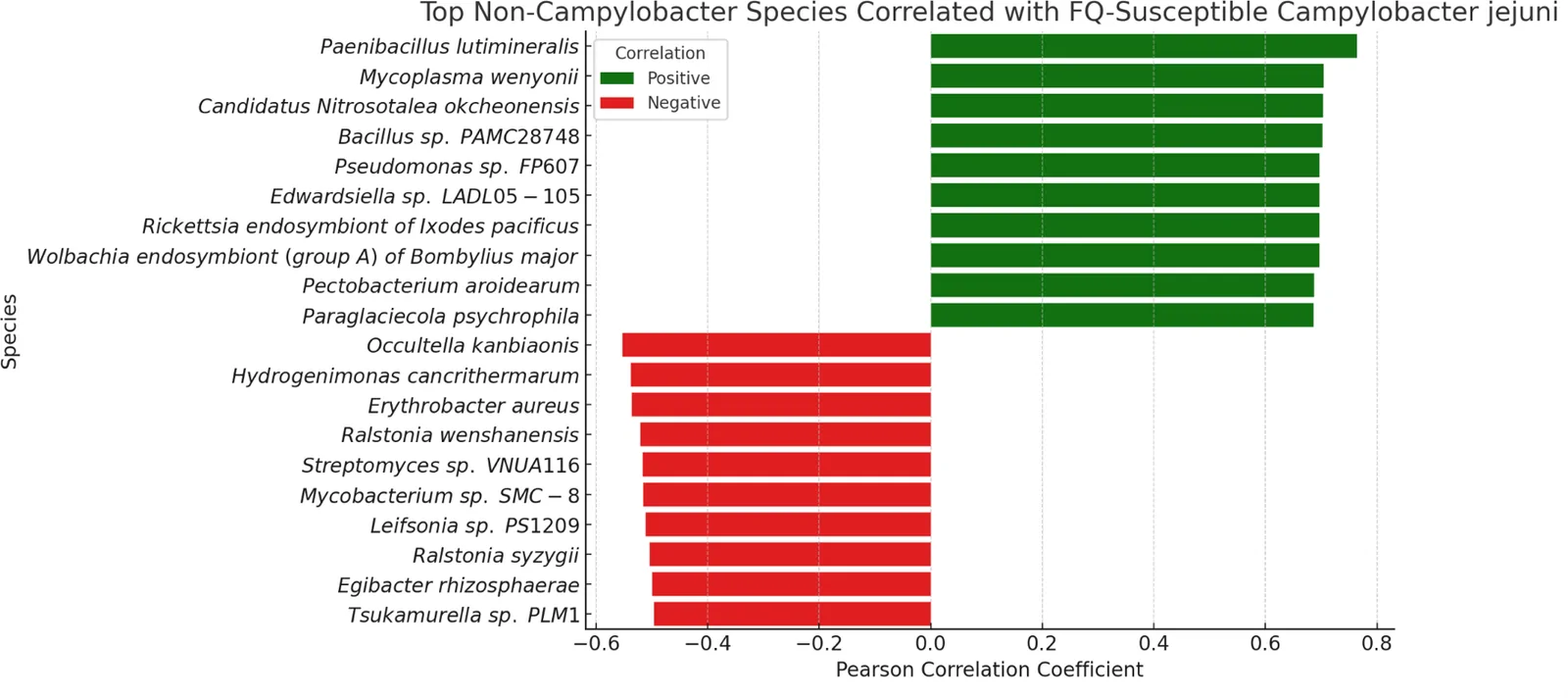
The top 10 most abundant microbial taxa correlated with FQ-susceptible *C. jejuni* in the cecal contents of the control group (not treated with ENRO). Analysis was done by Pearson’s correlation coefficient test of the shotgun metagenomics data from across all sampling time points.

#### Cecal microbiota members correlated with FQ-resistant *C. jejuni*

To examine potential associations between the cecal microbiota and FQ-resistant *C. jejuni*, Pearson’s correlation analysis was conducted between the abundances of *C. jejuni* and other bacterial species in the cecal contents of birds in the ENRO treatment group using the metagenomics data combined for all the sampling time points. Among the top 10 positively correlated species (Fig. 15) were *Clostridioides* sp. *ES-S-0010-02* (correlation coefficient = 0.715), *Olleya* sp. *Bg11-27* (correlation coefficient = 0.698), and *Enterococcus cecorum* (correlation coefficient = 0.649). Of the top 10 negatively correlated species (Fig. 15), *Ferrimonas* sp. *YFM* (correlation coefficient = −0.628), *Paenibacillus algicola* (correlation coefficient = −0.602), and *Acidaminococcus fermentans* (correlation coefficient = −0.597) were most notable, suggesting potential microbial antagonism or competition with FQ-resistant *C. jejuni* population.

**Fig 15:**
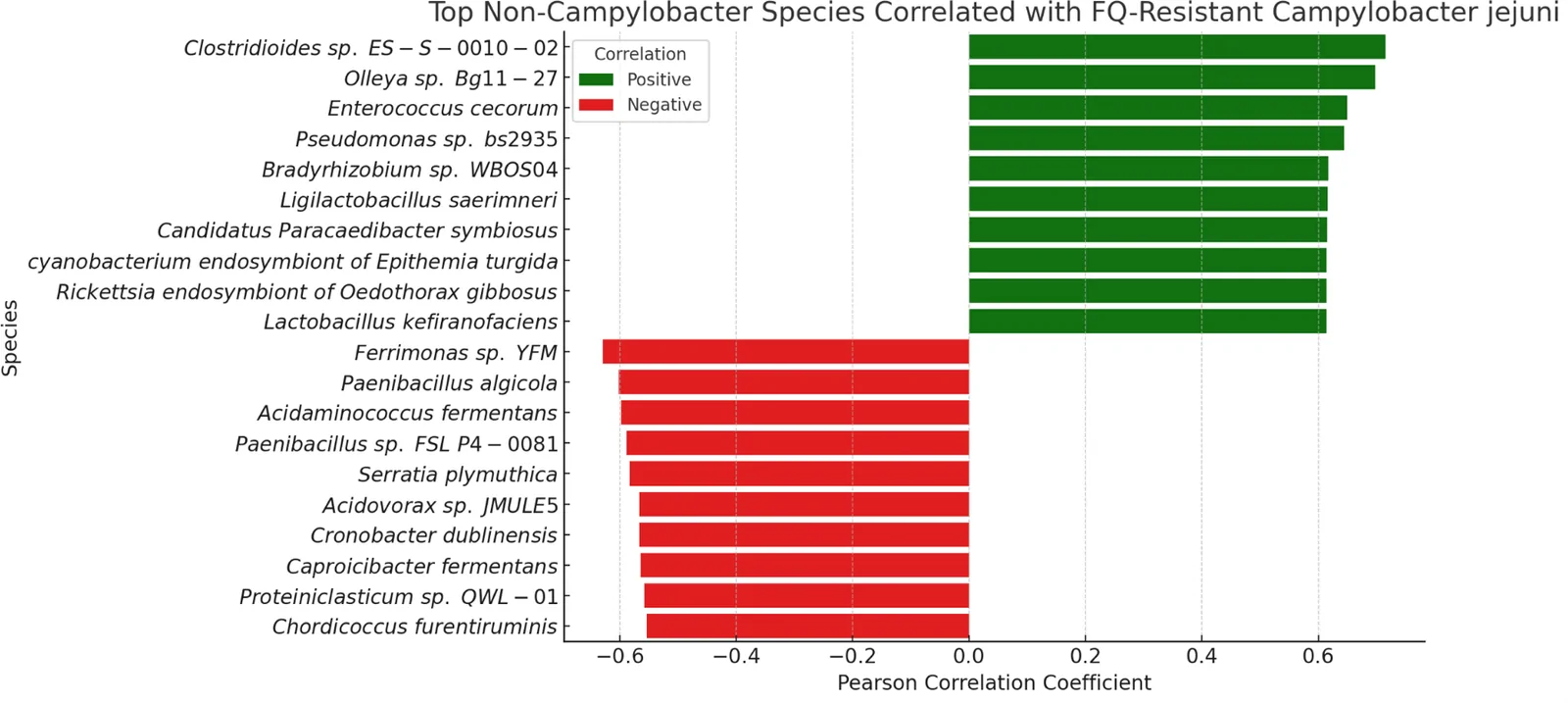
The top 10 most abundant microbial taxa correlated with FQ-resistant *C. jejuni* in the cecal contents of the ENRO treatment group. Analysis was done by Pearson’s correlation coefficient test of the shotgun metagenomics data from across all the sampling time points.

#### Microbial correlates of phenethylamine accumulation in the chicken cecum

To identify the gut microbiota members that may potentially be involved in the production or modulation of phenethylamine (the most differentially/positively upregulated metabolite in response to the ENRO treatment, Table 2), we performed correlation analysis between the metagenomics-based bacterial species abundance and the metabolomics-based phenethylamine levels in the cecal contents using three datasets (i.e., control group only, ENRO treatment group only, and both groups combined across all sampling time points) (Fig. 16). In the control group, phenethylamine showed moderate positive associations with *Streptomyces niphimycinicus* (r = 0.56), *Haladaptatus* sp. YSMS36 (r = 0.50), *Staphylococcus* sp. IVB6238 (r = 0.50), and *Corynebacterium* sp. CNCTC7651 (r = 0.49), while the strongest negative associations included *Paracoccus* sp. S3-43 (r = −0.57) and several taxa near r ≈ −0.56 (e.g., *Streptomyces* sp. Go40/10, *Azotobacter chroococcum*, *Novosphingobium* sp. 9), suggesting that no single species dominates the phenethylamine-linked signature (Fig. 16). In contrast, the ENRO-treatment group exhibited much stronger positive correlations overall, led by *Streptomyces* sp. 604F (r = 0.77), *Sulfolobus* sp. S-194 (r = 0.72), *Agrobacterium rubi* (r = 0.71), *Flavobacterium davisii* (r = 0.70), and *Borreliella carolinensis* (r = 0.69), whereas the notable negative associations were observed for *Acidithiobacillus* sp. “AMD consortium” (r = −0.63) and *Neorickettsia risticii* (r = −0.58), followed by several Firmicutes/Actinobacteria members (e.g., *Enterococcus raffinosus*, r = −0.55) (Fig. 16). When both groups combined, *Borreliella carolinensis* (r = 0.735) and *Wolbachia* endosymbiont of *Bemisia tabaci* (r = 0.730) were the top positively correlated taxa, with additional positive associations with *Stenotrophomonas* sp. WZN-1 (r = 0.601) and several environmental/food-associated taxa (e.g., *Aureibaculum algae*, r = 0.573), while the top negative correlates included *Erwinia* sp. E602 (r = −0.483) and *Streptosporangium roseum* (r = −0.442) (Fig. 16).

**Fig 16:**
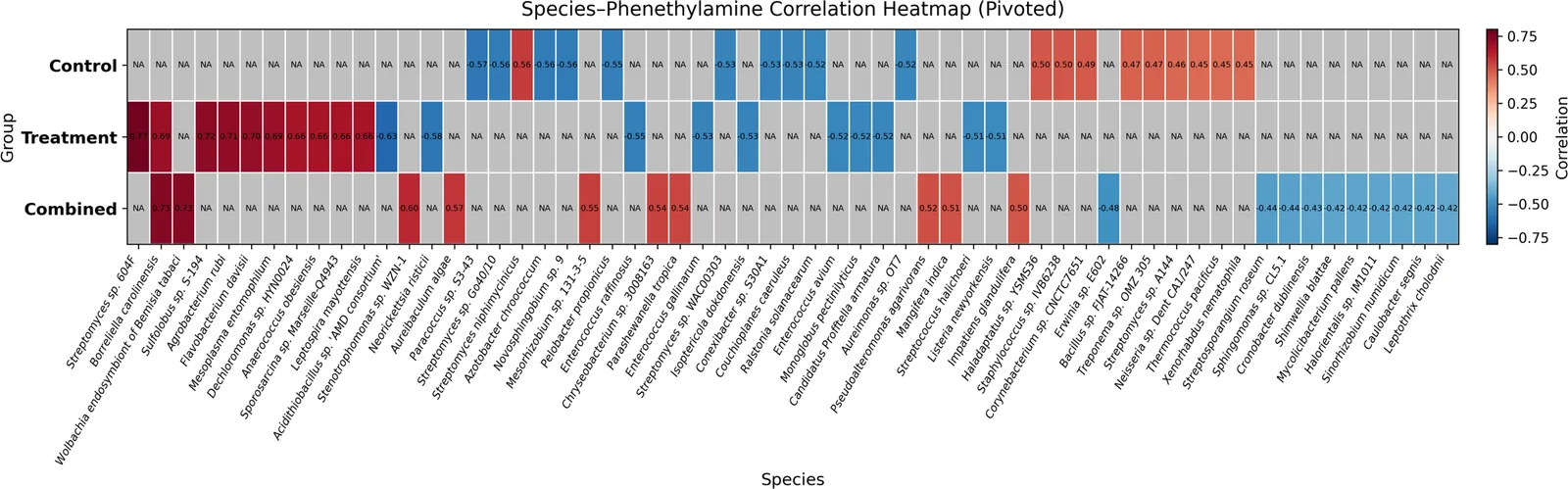
Heat map showing Pearson’s correlation coefficients for bacterial species abundance and phenethylamine levels in the control group, ENRO treatment group, and both groups combined. Significant correlations are colored in red (positive) or blue (negative). Only statistically significant (*P* value < 0.05) correlation coefficients are shown. Coefficients range from −1 to 1, with 1 representing the highest positive correlation and −1 representing the highest negative correlation.

#### Functional gene evidence for phenethylamine biosynthesis in chicken cecum in response to ENRO treatment

Functional annotation of metagenomic assemblies identified two key decarboxylase genes involved in phenethylamine biosynthesis: aromatic L-amino acid decarboxylase, which is the key enzyme catalyzing the decarboxylation of phenylalanine to phenethylamine; and tyrosine decarboxylase, which can also decarboxylate phenylalanine to phenethylamine to a lesser extent, especially when tyrosine (4-hydroxyphenylalanine) levels are depleted (56, 57). In this study, cecal contents of the ENRO-treated birds and the control birds harbored similar numbers of gene copies of aromatic L-amino acid decarboxylase (Table 3). However, even though the gene encoding tyrosine decarboxylase was present only in the control group during the earlier sampling time point (DPT-2), it was detected only in the ENRO treatment group during the later time points (DPT-7, DPT-12, and DPT-17), temporally aligning with the observed surge in phenethylamine levels (Table 3; also see Table 2 for details).

**TABLE 3:** Shotgun metagenomics-based gene counts of aromatic L-amino acid decarboxylase and tyrosine decarboxylase enzymes (potentially involved in phenethylamine biosynthesis) in cecal contents of chickens in the control and ENRO treatment groups across different time points

Time point	Group	Aromatic L-amino-acid decarboxylase gene counts	Tyrosine decarboxylase gene counts	Fold-change (E-DPT/C-DPT)^*a*^
DPT-2	Control	9	1	0.86
DPT-2	ENRO	7	0	
DPT-7	Control	9	0	14.92
DPT-7	ENRO	10	1	
DPT-12	Control	12	0	240.47
DPT-12	ENRO	13	1	
DPT-17	Control	11	0	186.86
DPT-17	ENRO	10	1	

^*a*^ Fold-change shows the ratio of the mean scaled intensity for phenethylamine between the ENRO treatment group (E) and the control group (C) (data derived from Table 2). DPT, day post-initiation of ENRO treatment.

## DISCUSSION

*Campylobacter*, a foodborne pathogen that spreads to humans through food-related pathways, is often subjected to selection pressure from antimicrobials used in food animal production. It has been shown that antibiotic use drives the evolution of antibiotic-resistant *Campylobacter* in the intestinal tract of broilers (17, 18). The gut microbiota plays pivotal roles not only for the digestion of food but also for maintaining the host’s overall health (33). Antibiotic treatment disrupts the gut microbiota of the host and subsequently affects the balance of microbiomes and metabolomic conditions (58). In the current study, we evaluated the effects of ENRO treatment on the intestinal microbiota compositions and metabolomes in the ceca of broiler chickens colonized with *C. jejuni*. The results showed that even though ENRO treatment given in drinking water transiently lowered *Campylobacter* colonization levels, it rapidly promoted the selection and propagation of FQ-resistant *Campylobacter*, which eventually replaced the FQ-susceptible population in the chicken intestine. Additionally, although ENRO treatment decreased the cecal microbiota diversity significantly, this returned to normal after the treatment was terminated, with no significant differences observed compared with the untreated controls. However, ENRO treatment resulted in significant structural changes in the cecal microbiota composition throughout the entire study. Finally, untargeted metabolomics also revealed significant metabolic changes in multiple pathways after ENRO treatment. Notably, one metabolite (phenethylamine) in the amino acid super pathway increased drastically after ENRO treatment. Collectively, these results support a model in which ENRO treatment both directly selects for resistant *C. jejuni* and transiently filters the gut microbial community and metabolic environment, potentially shaping the ecological context in which the resistant population persists.

The effect of antibiotic treatment on gut microbiome composition and function has been intensively studied, and it is widely recognized that the use of antibiotics decreases microbial diversity and alters the structure of microbiomes in both humans and animals (59, 60). Our results showed that the overall alpha diversity difference between the ENRO treatment and the control group was minimal, except for the phylogenetic diversity, where the control group had significantly higher Faith’s pd index compared with the ENRO treatment group (Fig. 2). However, when different sampling time points were considered (Fig. 3), the overall alpha diversity was significantly decreased soon after (DPT-2) the ENRO treatment (all four alpha diversity indices were significantly lowered in the ENRO treatment group compared with the control group). Observed features and Faith’s pd indices of the ENRO treatment group remained lower than the control group shortly after (on DPT-7) the cessation of the ENRO treatment. However, the alpha diversity difference between the two groups completely disappeared, starting from DPT-12 until the end of the study. Reduced alpha diversity shortly after cessation of the ENRO treatment, then full recovery, was also observed in a recent study, where different doses (low dose: 10 mg/kg b.w.; high dose: 100 mg/kg b.w.) of ENRO were given orally to 1-day-old broilers for 5 consecutive days, and cecal samples were collected to evaluate the effect of ENRO treatment on the microbiota when birds were 7-, 14-, and 21-days old (40). In particular, the untreated group had the highest microbial diversity on day 7, but there were no significant differences in microbial diversity among groups on days 14 and 21 (40). In contrast, Beyi et al. observed no significant changes in the fecal microbiota alpha diversity (observed features and Shannon indices) of calves treated with various doses of ENRO (36).

Our findings showed that ENRO treatment significantly altered the chicken cecal microbiota structure and composition (Fig. 4). The main drivers for the changes in the microbial structure were antibiotic treatment and the bird’s age, as revealed by the ANOSIM tests (Fig. 5). The dissimilarity rank value was significantly lower in the ENRO-treated group, confirming that antibiotic treatment reduces the complexity of microbial structure to a large extent. These results are in line with the finding by Elokil et al., who investigated the effect of ENRO treatment on the diversity of chicken gut microbiota and observed that the microbiota composition among samples of the control chicks was significantly different from that of the antibiotic-exposed chicks by ANOSIM analysis (61). Ma et al. also observed significant structural changes in the fecal microbial communities owing to different doses of antibiotic treatment and physical development stages when testing the effect of ENRO treatment on the cecal microbiome of broilers (40, 62).

The chicken cecal microbiota community comprises around 1,000 distinct bacterial species, categorized into two primary phyla, *Firmicutes* and *Bacteroidota*, along with two less predominant phyla, *Actinobacteria* and *Proteobacteria* (33). However, the top four abundant phyla identified in our study were *Firmicutes*, *Campylobacterota*, *Proteobacteria*, and *Actinobacteriota*, with *Campylobacterota* replacing *Bacteroidota* as the second most abundant phylum (Fig. 6). Notably, *Campylobacterota* was the second most abundant phylum in the ENRO treatment group and the fourth most abundant phylum in the control group. This may be explained by the fact that the chickens were inoculated with *C. jejuni* at 1 week of age. Even though the microbial community structure did not differ notably at the phylum level, the differences observed at the genus level were more pronounced (Fig. 6). Notably, the abundances of *Proteus*, *Blautia*, *Romboutsia*, *Erysipelotrichaceae_uncultured*, *Christensenellaceae_R-7_group*, *RF39*, *Candidatus_Arthromitus*, *Weissella*, *Butyricicoccus*, and *Clostridia_vadinBB60_group* were significantly decreased after ENRO treatment (Fig. 7). In contrast to our result, a previous study reported an increase of *Blautia*, *Weissella*, and *Erysipelotrichaceae* in broilers treated with ENRO and challenged with *Salmonella* Typhimurium (62). In a previous study, we investigated fecal microbiota transplantation (FMT) to assess the potential transfer of *Campylobacter* colonization resistance from the gut microbiota of *Campylobacter*-negative adult broilers (63). We observed notable decreases in the abundances of certain cecal microbiota members, such as *Proteus*, *Weissella*, and *Romboutsia*, following the FMT procedure. It is interesting to see that both FMT and ENRO treatment caused a decrease in these three bacterial genera. The only genus that showed an increase in abundance after ENRO treatment in the current study was the genus *Clostridium_innocuum_group*, and the increase of this same genus was also observed when broilers were fed ENRO in another study (62).

In this study, we analyzed the cecal metabolome of broilers treated with or without ENRO and colonized with *C. jejuni*. The fecal metabolome reflects, to a large extent, the gut microbiome and physiology of the host (64, 65). To the best of our knowledge, this is the first study that profiled the cecal metabolome response in chickens colonized with *C. jejuni* and treated with ENRO. Both the pathway- and metabolite-level analyses revealed that the ENRO treatment significantly altered the cecal metabolome. Lipids were the most commonly identified metabolites (Fig. 9), and lipid metabolism was the most altered metabolic pathway (Table 2). In total, 261 lipids with known structures were identified, among which 28 exhibited significantly different levels between the ENRO treatment group and the control group. Most of these differentially abundant lipids were elevated in the ENRO treatment group; however, significant levels of differences were only achieved when the combined data for the entire study, not at separate sampling time points, were accounted for. Of the 28 lipids that were significantly altered, 9 exhibited significant changes at one or more sampling times. The most markedly upregulated lipids by ENRO were choline phosphate and glycerophosphorylcholine (GPC), both belonging to the phospholipid metabolism sub-pathway (55). Phospholipids constitute the predominant proportion of cellular membrane lipids and play integral roles in cell signaling, cell metabolism, and even cell pathophysiology (66). The observed differences in the cecal content’s phospholipids could be an indication of altered production, utilization, or turnover of these molecules in the chicken gut in response to ENRO treatment following *C. jejuni* colonization. ENRO treatment also altered carnitine-related lipid metabolism, as shown by increased butyrylcarnitine (fold-change = 2.64) and decreased deoxycarnitine (fold-change = 0.45) in the ENRO-treated birds. Butyrylcarnitine is a short-chain acylcarnitine involved in acyl-group transport and fatty acid oxidation (67).

Deoxycarnitine is a precursor in L-carnitine biosynthesis and is closely linked to carnitine-dependent fatty acid metabolism (68). L-carnitine has important roles in poultry intermediary metabolism and fatty acid oxidation, and it serves several functions, including promoting growth, boosting immune function, providing antioxidant effects, and enhancing semen quality (69). The opposite changes in these two metabolites suggest that ENRO treatment may perturb the cecal carnitine/acylcarnitine metabolic network, potentially reflecting altered lipid utilization, short-chain acyl-CoA handling, or microbiota-associated transformation of carnitine-related substrates. Because the cecum is a major site of microbial fermentation in chickens, these changes may also be linked to antibiotic-induced shifts in microbial metabolism and short-chain fatty acid-related pathways. However, the source of these metabolites cannot be distinguished by untargeted metabolomics alone; thus, this result should be interpreted as evidence of ENRO-associated metabolic remodeling rather than a direct causal mechanism.

Another significantly altered sub-pathway within the lipid category is lysophospholipid. Several lysophospholipids, such as 2-stearoyl-GPE (18:0)* and 1-oleoyl-GPI (18:1), were significantly higher or trending higher in the ENRO-treated group compared with the control group at respective time points. Dietary supplementation of lysophospholipids was shown to increase *Lactobacillus* population, nutrient digestion, and growth and performance of broiler chickens (70). These data suggest that ENRO treatment might increase lysophospholipid levels in *Campylobacter*-colonized broiler chickens, which could influence the growth performance of birds.

Second to the lipid pathway, the amino acid pathway also underwent significant changes (Table 2). Significant alterations in aromatic amino acid (phenylalanine, tyrosine, and tryptophan) metabolism were observed. Specifically, the phenylalanine metabolite phenethylamine was significantly higher in the ENRO treatment group at all but the first sampling time points compared with the control group. Phenethylamine, with a significant overall fold-change of 115.38 between the ENRO and the control groups, was the most significantly elevated metabolite in the ENRO treatment group. In agreement with this finding, Ma et al. also observed significant enrichment of aromatic amino acid metabolites in broilers given ENRO in the drinking water for 5 consecutive days (40). A wide variety of species in the animal and plant kingdoms, including humans, produce phenethylamine (71). It is also produced by some bacteria, such as *Lactobacillus*, *Clostridium*, and *Pseudomonas*, and members of the *Enterobacteriaceae* family (72). Phenethylamine is a biogenic amine that serves as a precursor to various neurotransmitters and plays essential roles in regulating gastrointestinal motility and immune function (73–75). Its alteration in the cecal metabolome could have significant implications for the overall health and performance of the birds. One possible explanation for the increased phenethylamine levels could be attributed to the effects of ENRO treatment on the cecal microbiota composition. Antibiotics, including ENRO, are known to exert selective pressure on the gut microbiome, leading to changes in microbial populations (76). This alteration in the microbial community may promote the proliferation of specific bacteria capable of producing phenethylamine or enhancing the enzymatic activities responsible for its synthesis. Previous studies revealed that *Morganella morganii* produced significant quantities of phenethylamine that can readily cross the blood-brain barrier in mammals (65, 77). In another study involving a mouse model, it was observed that the human gut bacterium *Ruminococcus gnavus* had the ability to convert phenylalanine into phenethylamine through decarboxylation, and that an increase in fecal phenethylamine level was detected in germ-free mice colonized with *R. gnavus*, coinciding with impaired glucose tolerance and insulin sensitivity (78). Although we did not detect *R. gnavus*, *M. morganii* was detected via shotgun metagenomics in the current study. Thus, it is highly likely that the observed increase in phenethylamine level may be a direct consequence of antibiotic-induced changes in the gut microbiota. Another important aspect to consider is the interplay between the ENRO treatment and the challenge with *C. jejuni*. The combination of ENRO treatment and *Campylobacter* challenge may have led to a complex host-microbe interaction within the cecal environment, thus making the gut more favorable for the proliferation or metabolism of specific bacteria involved in phenethylamine production. In addition to the neurotransmitter effect, phenethylamine has been used for food processing. Lynnes et al. showed that processing beef with 150 mg/mL phenethylamine resulted in an 85% reduction in final *Escherichia coli* O157:H7 counts compared to the beef treated with ddH_2_O (75). Phenethylamine also exhibited a bactericidal effect against *Listeria monocytogenes* in both *in vitro* and various food models (79). In summary, the diverse impact of phenethylamine on both host and microbiota suggests that the increased phenethylamine levels observed in ENRO-treated chickens may have complex functional consequences, which remain to be determined in future studies.

Xenobiotics was the third most differentially altered metabolic pathway besides lipid and amino acid pathways. It is worth mentioning that sulfociprofloxacin was significantly higher during and shortly after the ENRO treatment period (DPT-2 and -7). Sulfociprofloxacin, which is also called ciprofloxacin piperazinyl-N4-sulfate, is a metabolite of ciprofloxacin (80). Thus, the detection of sulfociprofloxacin in the ENRO treatment group could be considered as additional evidence for the accuracy of the metabolomic profiling and quantification in the current study.

ENRO treatment promptly disrupted the gut microbial landscape, as evidenced by distinct early-time point profiles (DPT-2, DPT-7) between the ENRO treatment group and control group (Fig. 13). Over time (i.e., DPT-12 and DPT-17), the microbiota profiles between the two groups partially converged, a pattern consistent with antibiotic-driven transient dysbiosis, followed by ecological rebalancing. Notably, some taxa, such as *Flavonifractor plautii*, *Dysosmobacter welbionis*, and *Lachnoclostridium phocaeense*, remained dominant, whereas *Gordonibacter pamelaeae* (prevalent early in the control group) declined under treatment, hinting at shifts in functional capacity and microbial roles over time. *G. pamelaeae* was reported to catalyze the 21-dehydroxylation of biliary corticoids and convert them into progestins in the human gut, a class of sex hormones and neurosteroids involved in immune and metabolic regulation (81). This biochemical capability suggests that *G. pamelaeae* may contribute to host-microbe signaling pathways and modulation of intestinal immune response. Its marked reduction in ENRO-treated birds observed in the current study could therefore have downstream consequences on the gut hormonal milieu, potentially altering competitive dynamics within the microbial community and impacting metabolite networks, including those linked to phenethylamine production. While the direct role of *G. pamelaeae* in avian hosts remains to be confirmed, its known functional repertoire underscores the importance of considering microbial hormone metabolism when interpreting antibiotic-induced shifts in gut microbiota.

Correlation analysis revealed FQ-susceptible and FQ-resistant *C. jejuni* interact differently with other species in the chicken gut (Fig. 14 and 15). In the control birds, *Paenibacillus lutimineralis* and *Mycoplasma wenyonii* significantly co-occurred with susceptible *C. jejuni*, while taxa such as *Occultella kanbiaonis* showed inverse relationships. In contrast, FQ-resistant *C. jejuni* population in the ENRO-treated birds positively correlated with such taxa as *Clostridioides* and *Enterococcus cecorum*, which are organisms often associated with antibiotic-adapted gut communities (82, 83). Such shifts in gut microbiota suggest that ENRO treatment fosters microbial environments conducive to selection and propagation of FQ-resistant *C. jejuni*, potentially through reduced competition or altered niche occupancy.

In the current study, an interesting finding was the remarkably elevated phenethylamine levels in the ENRO-treated birds at all but the first sampling time point (Table 2). This metabolic shift aligns temporally with the detection of tyrosine decarboxylase gene in the ENRO-treated birds and the absence of the gene in the control birds (Table 3), suggesting functional enrichment toward aromatic amine (e.g., phenethylamine) production. Meanwhile, counts of aromatic L-amino acid decarboxylase gene (the primary gene involved in catalyzing decarboxylation of phenylalanine to phenethylamine) remained comparable between the groups. The co-occurrence of tyrosine decarboxylase gene and specific non-*Campylobacter* taxa positively correlated with phenethylamine supports the notion that ENRO-induced microbiota remodeling enhances microbial capacity for phenethylamine biosynthesis in the chicken gut, thereby reshaping metabolite profiles in such an environment (Fig. 16).

The species and phenethylamine correlation analysis indicates that phenethylamine accumulation is linked to different microbial signatures depending on treatment context, with stronger and more coherent associations under ENRO exposure. In the control group, correlations were only moderate (e.g., *Streptomyces niphimycinicus*, r = 0.56; most others |r| ≤ ~0.57), suggesting baseline phenethylamine variation likely reflects multiple community and host factors rather than a single dominant taxon (Fig. 16). In contrast, ENRO-treated samples showed markedly stronger positive correlations (up to *Streptomyces* sp. 604F, r = 0.77) and stronger negative correlates (e.g., *Acidithiobacillus* sp. “AMD consortium,” r = −0.63), consistent with antibiotic-driven ecological filtering that amplifies taxa most tightly linked to phenethylamine-associated metabolic states (Fig. 16). The combined data set highlights taxa whose associations persist across conditions, notably *Borreliella carolinensis* (r = 0.735) and *Wolbachia* endosymbiont of *Bemisia tabaci* (r = 0.730), nominating them as candidates that may contribute to, or consistently co-occur with, phenethylamine accumulation under ENRO-induced microbiome restructuring (Fig. 16).

Overall, the integrated analysis undertaken in the current study underscores the dual impact of ENRO treatment: (i) it disrupts gut microbial structure and selectively enriches functional traits tied to aromatic amine metabolism, possibly favoring colonization by FQ-resistant *C. jejuni*, and (ii) emergence of phenethylamine, a biogenic amine with known bioactive properties, may further alter gut physiology and community interactions, indicating that antibiotic use can drive complex biochemical consequences beyond mere microbial depletion. Future research should experimentally validate the phenethylamine-producing roles of candidate taxa and explore their interactions with resistant pathogens to inform precision microbiome modulation. A limitation of the current study is that the microbiota–metabolite–*Campylobacter* relationships were inferred mainly from correlation analyses. Therefore, the results primarily identify associations between candidate taxa and/or metabolites that may be linked to ENRO treatment and FQ-resistant *C. jejuni* expansion, rather than establishing direct causality, microbial competition, or metabolite-mediated promotion of resistance. Future studies using targeted metabolite supplementation, bacterial isolation, gnotobiotic or microbiota-transfer models, and *in vitro* co-culture assays will be needed to validate these potential interactions.

### Conclusion

This study provides an integrated multi-omics perspective on how ENRO administration alters the chicken cecal microbiota, its functional gene repertoire, and associated metabolite profiles. 16S rRNA gene and shotgun metagenomics sequencing revealed distinct taxonomic shifts between the control and ENRO-treated broiler chickens, with notable enrichment of specific bacterial taxa in the presence of the antibiotic. Correlation analyses identified both co-occurring and antagonistic microbiota members linked to FQ-susceptible *C. jejuni* and FQ-resistant *C. jejuni*, as well as to phenethylamine accumulation, which markedly increased in the ENRO-treated birds. Functional annotation indicated that while the aromatic L-amino acid decarboxylase gene was present in both groups at comparable counts throughout the study regardless of phenethylamine levels observed, the differential detection of the tyrosine decarboxylase gene in the ENRO group vs the control group temporally coincided well with the phenethylamine levels found in the respective groups at specific time points. These findings suggest that ENRO treatment not only selects for resistant *C. jejuni* populations, but is also associated with compositional and functional changes in the chicken gut microbiota and altered metabolite profiles, including phenethylamine accumulation. Furthermore, this work highlights the interplay between antibiotic-driven microbial selection, metabolic output, and potential impacts on host physiology, offering novel insights into the consequences of antimicrobial use in poultry production.

## Data Availability

Data are available upon request. The 16S rRNA sequence data were submitted to the NCBI SRA database under BioProject PRJNA1490980.
